# CoP/Fe‐Co_9_S_8_ for Highly Efficient Overall Water Splitting with Surface Reconstruction and Self‐Termination

**DOI:** 10.1002/advs.202204742

**Published:** 2022-10-21

**Authors:** Xinhong Chen, Yumeng Cheng, Yunzhou Wen, Yaya Wang, Xiao Yan, Jun Wei, Sisi He, Jia Zhou

**Affiliations:** ^1^ State Key Lab of Urban Water Resource and Environment School of Science Harbin Institute of Technology Shenzhen Shenzhen 518055 PR China; ^2^ School of Chemistry and Chemical Engineering Harbin Institute of Technology Harbin 150001 PR China; ^3^ Research Institute of Information Technology Shenzhen Institute of Information Technology Shenzhen 518172 PR China; ^4^ Shenzhen Key Laboratory of Flexible Printed Electronics Technology Center School of Materials Science and Engineering Harbin Institute of Technology Shenzhen 518055 PR China

**Keywords:** density functional theory (DFT) calculations, membrane electrode assembly (MEA) electrolyzer, oxygen evolution reaction (OER) electrocatalysts, surface reconstruction, water splitting

## Abstract

Highly efficient electrochemical water splitting is of prime importance in hydrogen energy but is suffered from the slow kinetics at the anodic oxygen evolution reaction. Herein, combining the surface activation with the heterostructure construction strategy, the CoP/Fe‐Co_9_S_8_ heterostructures as the pre‐catalyst for highly efficient oxygen evolution are successfully synthesized. The catalyst only needs 156 mV to reach 10 mA cm^−2^ and keeps stable for more than 150 h. Inductively coupled plasma optical emission spectrometry, in situ Raman spectroscopy and density functional theory calculations verify that the introduction of Fe can promote the formation of highly active Co(IV)–O sites and lead to a self‐termination of surface reconstruction, which eventually creates a highly active and stable oxygen evolution catalytic surface. Besides, the catalyst also demonstrates high hydrogen evolution reaction activity with an overpotential of 62 mV@10 mA cm^−2^. Benefiting from its bifunctionality and self‐supporting property, the membrane electrode assembly electrolyzer equipped with these catalysts achieves high overall water splitting efficiency of 1.68 V@1 A cm^−2^.

## Graphical abstract

By combining surface activation with a heterostructure construction strategy, a CoP/Fe‐Co_9_S_8_ heterostructure is successfully constructed as a high‐efficiency electrocatalyst. Benefiting from the zero‐gap architecture and the highly efficient performance, the membrane electrode assembly (MEA) electrolyzer only needs 1.68 V to reach 1 A cm^−2^, achieving low‐cost and high‐efficiency overall water splitting.

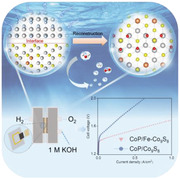

## 1 Introduction

Hydrogen energy is currently considered to be one of the most promising renewable energy resources to substitute traditional fossil energy.^[^ ^1^, ^2^, ^3^ ^]^ High‐efficiency water decomposition driven by renewable electricity is an effective way to produce hydrogen.^[^ ^4^, ^5^ ^]^ However, the anodic oxygen evolution reaction (OER) and cathodic hydrogen evolution reaction (HER) of electrochemical water splitting both suffer from unfavorable thermodynamics and slow kinetics, especially the multi‐step proton/electron transfer process of OER, which greatly hinders the overall efficiency of water electrolysis.^[^ ^6^, ^7^ ^]^ At present, the commercialized Ir/Ru‐based OER catalyst is facing the bottleneck of industrial application due to its scarcity and high price.^[^ ^8^, ^9^ ^]^ Therefore, it is urgent to develop non‐noble‐metal materials with high activity, long‐term stability, and low cost as alternative OER catalysts.

Recently, cobalt‐based materials are considered promising electrocatalysts for water splitting because of their low cost, high activity, and natural abundances, such as cobalt‐based phosphides,^[^ ^10^, ^11^ ^]^ sulfides,^[^ ^12^, ^13^ ^]^ oxides,^[^ ^14^, ^15^ ^]^ carbides^[^ ^16^, ^17^ ^]^ and nitrides.^[^ ^18^, ^19^ ^]^ Among them, heterojunction cobalt‐based catalysts are widely used in the field of water electrolysis due to the interaction of electrons at the interface. However, most research only pays attention to the structure‐activity relationship of these heterojunction cobalt‐based catalysts, ignoring the source of their activity, especially the surface of sulfide, phosphide, and nitride catalysts will be converted into metal hydroxide/oxide during the reaction.^[^ ^11^, ^12^ ^]^ Current studies have shown that such materials, known as pre‐catalysts, will undergo surface reconstruction during the OER process, thus transforming into the actual active phase.^[^ ^20^, ^21^ ^]^ These reconstructed catalysts usually display more outstanding OER catalytic performance than pre‐catalysts and pure oxides/hydroxides. By utilizing the in situ reconstruction of the surface, a higher specific surface area of the materials can be obtained, and the local atomic structures and electronic structures of the active sites can be regulated, thereby optimizing the adsorption and desorption energetics of the reaction intermediates and accelerating the OER process.^[^ ^22^, ^23^ ^]^

The activity and stability of catalysts will be affected by the reconstruction strategy and reconstruction depth. The conjugation of different reconstruction strategies, such as surface activation, heterostructure construction, defect engineering, ionic doping, partial dissolution, and deep reconstruction, may lead to a more active catalytic surface.^[^ ^24^, ^25^ ^]^ On the other hand, excessive reconstruction may destroy the structural stability of the catalysts due to the serious dissolution of components, resulting in the loss of catalysts’ stability.^[^ ^26^ ^]^ Therefore, the depth of reconstruction should be tuned to inhibit the dissolution of active species and eventually obtain an OER catalytic interface with efficiency and stability.^[^ ^27^ ^]^

In this work, we combined surface activation with a heterostructure construction strategy to synthesize the CoP/Co_9_S_8_ heterostructure interface, and further introduced Fe doping to regulate the reconstruction depth, thereby constructing CoP/Fe‐Co_9_S_8_ pre‐catalyst for OER. The catalyst displayed an ultra‐low overpotential of 156 mV at 10 mA cm^−2^ in the alkaline electrolyte, twice the mass‐specific activity of commercial RuO_2_, and remained stable after 150 h of continuous electrolysis. Electrochemical analysis, inductively coupled plasma optical emission spectrometry (ICP‐OES), and in situ Raman spectroscopy demonstrated that the introduction of Fe promoted the surface reconstruction of the catalyst and formed highly active Co(IV)‐O sites. Meanwhile, the combination of Fe and S could lead to self‐termination of surface reconstruction, inhibit the loss of P and S, and form a stable high valence Co active center. Density functional theory (DFT) calculation clarified that the high valence Co site can optimize the adsorption energy of OER intermediates, thus improving the activity of OER. Moreover, the pre‐catalyst before reconstruction exhibited excellent HER activity (with an overpotential of 62 mV at 10 mA cm^−2^), forming a bifunctional catalyst with both OER and HER activities. We further assembled a membrane electrode assembly (MEA) alkaline water electrolyzer with these catalysts. The cell voltage of this electrolyzer to reach 1 A cm^−2^ is only 1.68 V, achieving a low‐cost and high‐efficiency overall water splitting device.

## 2 Results and Discussion

### 2.1 Synthesis and Morphology of the CoP/Fe‐Co_9_S_8_ Electrode

**Figure 1:**
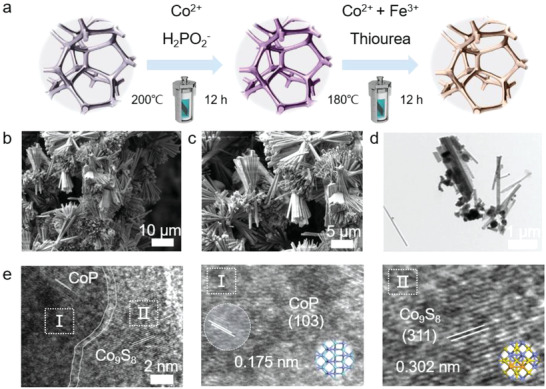
Synthesis and characterization of the materials. a) Illustration for preparing CoP/Fe‐Co_9_S_8_ electrode. Phase and composition analyses of the powder sample CoP/Fe‐Co_9_S_8_ for b,c) SEM images at different magnifications and d,e) TEM images at different magnifications.

We synthesized the heterostructure catalysts by a two‐step hydrothermal method (**Figure** **1**a). In the first step, by regulating the reaction solution composed of cobalt sulfate and sodium hypophosphite, the cactus‐like CoP nanoarray precursor was hydrothermally grown on nickel foam (NF) (Figures S1 and S2, Supporting Information). Then, by controlling the composition of the solution containing cobalt nitrate, iron sulfate, and thiourea, the precursor was further vulcanized at an appropriate temperature to obtain a heterostructure catalyst (denoted as CoP/Fe‐Co_9_S_8_). Scanning electron microscope (SEM) images (Figure 1b,c) show that the heterostructure CoP/Fe‐Co_9_S_8_ electrode has a 3D nanorod morphology with a large specific surface area. In contrast, the Fe‐Co_9_S_8_ sample grown directly on NF is composed of many nanoparticles with a lower surface area (Figure S3, Supporting Information). The X‐ray diffraction peaks of the powder sample for the catalyst confirm that they are composed of CoP and Co_9_S_8_ phases (Figure S4, Supporting Information).^[^ ^28^, ^29^ ^]^ The proportion of each element is consistent with that of CoP and Co_9_S_8_, and the trace amount of iron further confirms the successful doping of Fe, as verified by the energy dispersive X‐ray spectroscopy (EDS) of various catalysts (Figure S5, Supporting Information).

To further demonstrate the formation of the heterostructure, transmission electron microscopy (TEM) was employed to characterize the powder sample for the catalyst. Nanorods with a diameter of ≈200 nm are observed on the surface of the substrate (Figure 1d). The high‐resolution TEM images (HR‐TEM, Figure 1e) at the edge of the nanorod show a clear interface between crystallized CoP and Co_9_S_8_. The enlarged views of the crystal plane in regions I and II indicate that the lattice fringe with d‐spacing of 0.175 and 0.302 nm, corresponds to the CoP (103) planes and Co_9_S_8_ (311) planes, respectively.^[^ ^30^, ^31^, ^32^ ^]^ Through high‐angle annular dark‐field scanning TEM (HAADF‐STEM) EDS elemental mapping, the uniform distribution of Co, P, Fe, and S in the CoP/Fe‐Co_9_S_8_ were confirmed (Figure S6, Supporting Information). Therefore, it is proved that the heterostructure of CoP/Fe‐Co_9_S_8_ has been successfully constructed.

### 2.2 Electrochemical Catalytic Performance

**Figure 2:**
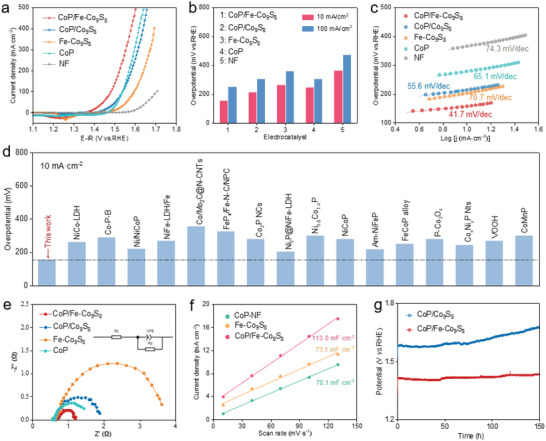
OER catalytic performance of electrocatalyst in 1 m KOH alkaline medium. a) Polarization curves of the CoP/Fe‐Co_9_S_8_ and other catalysts at 5 mV s^−1^. b) overpotentials at 10 and 100 mA cm^−2^. c) Steady‐state Tafel slope. d) Comparison of OER performance. e) Nyquist plots at 1.53 V versus RHE. f) ECSA estimated by C_dl_ values. g) The time‐potential curve under the constant current density of 10 mA cm^−2^.

We then evaluated the OER performance of CoP/Fe‐Co_9_S_8_ in a three‐electrode system using 1 m KOH solution as the electrolyte. Linear sweep voltammetry curves (**Figure** **2**a; Figures S7–S9, Supporting Information) reveal the catalytic activities of CoP/Fe‐Co_9_S_8_ with the control samples of CoP/Co_9_S_8_, Fe‐Co_9_S_8_, CoP, and NF. CoP/Fe‐Co_9_S_8_ displays the best OER catalytic activity with a low overpotential of 156 mV at the current density of 10 mA cm^−2^. The OER performance of CoP/Fe‐Co_9_S_8_ is much lower than that of CoP/Co_9_S_8_ (214 mV), Fe‐Co_9_S_8_ (266 mV), CoP (249 mV), and NF (365 mV) (Figure 2b). The steady‐state Tafel plots show that CoP/Fe‐Co_9_S_8_ exhibits a Tafel slope of 41.7 mV dec^−1^, which is smaller than that of CoP/Co_9_S_8_ (55.6 mV dec^−1^), Fe‐Co_9_S_8_ (70.7 mV dec^−1^), CoP (65.1 mV dec^−1^) and NF (74.3 mV dec^−1^), indicating a faster OER kinetics on CoP/Fe‐Co_9_S_8_ (Figure 2c; Figure S10, Supporting Information). It is worth noting that the OER performance of CoP/Fe‐Co_9_S_8_ electrocatalyst is superior to that of previously reported Co‐based and Fe‐based electrocatalysts (as shown in Table S1, Supporting Information; Figure 2d), which may originate from the dual regulation of Fe doping and the heterogeneous active interface. In addition, the electrochemical impedance spectroscopy confirmed that the CoP/Fe‐Co_9_S_8_ has the smallest charge transfer resistance (*R*_ct_) during OER (Figure 2e; Table S2, Supporting Information), which proves the small energy barrier of rapid electron transport. Then, the double‐layer capacitance (*C*_dl_) was measured by cyclic voltammetry (CV) curves (Figure S11, Supporting Information) to evaluate the electrochemical active surface area (ECSA) of these electrocatalysts (Figure 2f). The CoP/Fe‐Co_9_S_8_ displays the highest C_dl_ value of 113.0 mF cm^−2^, indicating more active sites, which is conducive to promoting electrocatalytic OER. The number of real active sites of different catalysts was further detected by the redox peak method (Figure S12, Supporting Information), and the results showed that CoP/Fe‐Co_9_S_8_ had more active sites. The iR‐corrected polarization curves in terms of the current density normalized with ECSA for the CoP/Fe‐Co_9_S_8_ have been tested to compare the intrinsic catalytic activity of the catalysts (Figure S13, Supporting Information), indicating that the intrinsic activity of the CoP/Fe‐Co_9_S_8_ exceeded that of control samples. The above results showed that the improvement of OER activity in CoP/Fe‐Co_9_S_8_ is due to both the increase of accessible active site numbers and the improvement of the intrinsic activity of the active sites.

The long‐term durability was further traced by the CV and chronopotentiometry (CP) methods in KOH electrolytes. The stability test results (Figure 2g) showed that the OER overpotential for the CoP/Fe‐Co_9_S_8_ increased from 156 to 204 mV after reaction in 1 m KOH for 150 h under the constant current density of 10 mA cm^−2^. The degradation rate of catalytic performance for the CoP/Fe‐Co_9_S_8_ is only 0.32 mV h^−1^, which confirms the excellent stability of the catalyst. While the Fe‐free sample (CoP/Co_9_S_8_) degraded rapidly, with a degradation rate of 1.49 mV h^−1^ – five times the CoP/Fe‐Co_9_S_8_, which showed that the Fe‐doping could not only improve the OER activity but also contribute to the stability. The OER polarization curve after 2000 continuous CV scans showed little degradation, which further verified the stability of CoP/Fe‐Co_9_S_8_ (Figure S14, Supporting Information). In the post‐OER XPS spectra of catalysts, the surface chemical state of CoP/Fe‐Co_9_S_8_ hardly changed after OER except for a small amount of P was lost (Figure S15, Supporting Information). The HR‐TEM image showed that some CoOOH phase was generated at the catalyst surface after OER (Figure S16, Supporting Information). While the bulk CoP/Fe‐Co_9_S_8_ was not changed significantly and the general morphology was kept unchanged after stability tests (Figure S17, Supporting Information). The results showed that the CoP/Fe‐Co_9_S_8_ pre‐catalyst underwent a surface reconstruction under OER conditions, while the bulk property (mainly the Co_9_S_8_ phase) was retained.

### 2.3 In Situ Investigation of the Surface Reconstruction and OER Mechanism

**Figure 3:**
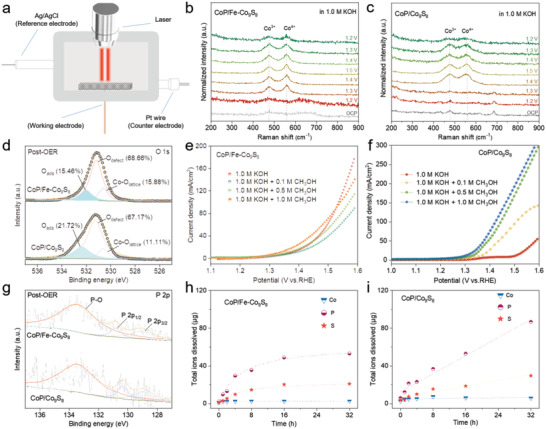
Study of the surface reconstruction on CoP/Fe‐Co_9_S_8_ electrode during OER. a–c) In situ Raman spectra of OER on CoP/Fe‐Co_9_S_8_ in 1 m KOH. d) XPS spectra of O 1*s* after alkaline OER on CoP/Fe‐Co_9_S_8_ and Fe‐Co_9_S_8_. e,f) MOR performance test on CoP/Fe‐Co_9_S_8_ in 1 m KOH. g) XPS spectra of P 2*p* after alkaline OER on CoP/Fe‐Co_9_S_8_ and Fe‐Co_9_S_8_. h,i) ICP‐OES test after alkaline OER on CoP/Fe‐Co_9_S_8_.

To explore the mechanism of the high OER activity of CoP/Fe‐Co_9_S_8_, we studied the reconstruction from CoOOH to CoO_2_ on CoP/Fe‐Co_9_S_8_ with CoP/Co_9_S_8_ as the control sample by CV measurement. As shown in Figure S18 (Supporting Information) the CV behaviors showed that CoP/Fe‐Co_9_S_8_ possess a lower Co^3+^/Co^4+^ conversion potential than that of CoP/Co_9_S_8_, indicating the favorable conversion of Co^3+^ to active Co^4+^ on the surface.^[^ ^12^ ^]^ Besides, we further traced the catalyst surface evolution by obtaining in situ electrochemical Raman spectra of catalysts under different electrode potentials (**Figure** **3**a–c). There are two main Raman peaks at 471 and 567 cm^−1^, which can be attributed to Co^3+^ and Co^4+^, respectively.^[^ ^33^, ^34^, ^35^ ^]^ The Raman peak corresponding to Co^4+^ appeared in the spectrum of CoP/Fe‐Co_9_S_8_ when the applied potential increased to 1.3 V versus RHE, while it did not appear in the spectrum of CoP/Co_9_S_8_ until the applied potential reached 1.4 V. It was proved that the Co phase in CoP/Fe‐Co_9_S_8_ was easier to be oxidized Co^4+^ in the OER process compared to CoP/Co_9_S_8_, indicating that the surface reconstruction was promoted by the Fe incorporation. The post‐OER XPS spectrum of O 1*s* showed that CoP/Fe‐Co_9_S_8_ had more abundant lattice oxygen (Co–O_lattice_), which may come from Co(IV)O_2_ (Figure 3d). While CoP/Co_9_S_8_ possesses more adsorbed oxygen species (O_ads_), which may attribute to Co(III)OOH, consistent with the results of Raman spectroscopy.^[^ ^36^, ^37^, ^38^, ^39^, ^40^ ^]^ The methanol oxidation reaction (MOR) was also conducted to further detect the coverage of surface hydroxyl on CoP/Fe‐Co_9_S_8_. As shown in Figure 3e,f, the CoP/Fe‐Co_9_S_8_ is inert to the MOR, which indicates low OH* coverage on the surface; while CoP/Co_9_S_8_ shows higher MOR activity, indicating a much higher OH* coverage.^[^ ^41^, ^42^, ^43^ ^]^ The results successfully prove that the introduction of Fe promoted the surface reconstruction of the catalyst and formed highly active Co(IV)–O sites.

To further evaluate the reconstruction depth during OER, we carefully examined the XPS spectrum of CoP/Fe‐Co_9_S_8_ and CoP/Co_9_S_8_ before and after the long‐term OER process (Figure 3g; Figure S15, Supporting Information). The P element at the surface of CoP/Co_9_S_8_ showed a more serious loss, while CoP/Fe‐Co_9_S_8_ displayed a more amount of Fe–S bond during catalytic OER compared to CoP/Fe‐Co_9_S_8_. The combination of Fe and S may prevent the loss of anions during OER.^[^ ^44^ ^]^ The element leaching process was further examined by the ICP‐OES (Figure 3h,i).^[^ ^45^, ^46^ ^]^ The P element in the CoP/Fe‐Co_9_S_8_ catalyst was rapidly leached at the beginning of the reaction and stabilized after ≈16 h. The S element dissolved in a small amount at the beginning of the reaction, while the Co ion hardly changed. In contrast, both S and P showed significant leaching in CoP/Co_9_S_8_. This proves that the doping of Fe could lead to self‐termination of surface reconstruction, inhibit the loss of P and S, and form a stable high valence Co active structure. To quantitively verify the depth of reconstruction, we carried out composition analysis at different depths for the catalyst after 24 h OER reaction using Ar‐ion etching XPS depth analysis (Figure S19, Supporting Information). It shows that the atomic contents of P and S increased along with the etching depth, and kept stable after the etching depth reaches 10 nm. Which verified that the top‐10 nm of the catalyst reconstructed and formed active phases. Hence, the incorporation of Fe into the CoP/Co_9_S_8_ heterostructure could promote the formation of high valence Co sites and terminate the reconstruction of the surface, thus leading to high OER activity and stability.

### 2.4 DFT Calculations

**Figure 4:**
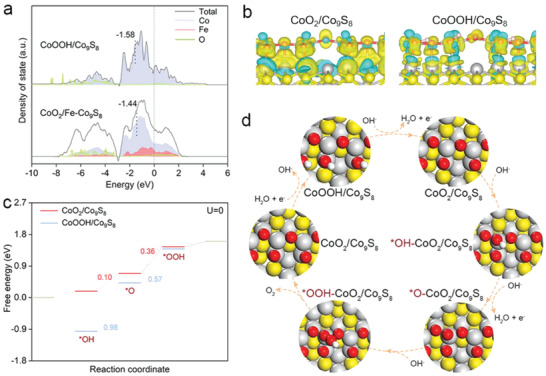
DFT calculations. a) The density of states (DOS) of Co‐3d, Fe‐3d, and O‐2p on CoO_2_/Fe‐Co_9_S_8_ and CoOOH/Co_9_S_8_ surfaces. b) The charge density differences for CoO_2_/Co_9_S_8_ and CoOOH/Co_9_S_8_ surfaces. c) Gibbs free energy illustration by CoO_2_/Co_9_S_8_ and CoOOH/Co_9_S_8_ during the OER process. d) Proposed OER mechanism of CoO_2_/Co_9_S_8_.

We carried out DFT calculations on CoO_2_/Fe‐Co_9_S_8_ and CoOOH/Co_9_S_8_ to further illustrate how Co(IV) promotes the OER activity (Figures S20–S22, Supporting Information). The *d*‐band partial density of states (PDOS) of Co atoms in CoO_2_/Fe‐Co_9_S_8_ and the *p*‐band PDOS of O atoms were analyzed to predict the adsorption intensity of oxygen intermediate species in the OER process (**Figure** **4**a). Compared with CoOOH/Co_9_S_8_, the calculated *d*‐band center of Co atoms in CoO_2_/Fe‐Co_9_S_8_ is closer to the Fermi energy level, revealing the stronger adsorption capacity of oxygen intermediate species on CoO_2_/Fe‐Co_9_S_8_.^[^ ^47^, ^48^, ^49^ ^]^ In addition, the charge density differences showed that the Co atoms had a more positive charge in CoO_2_/Co_9_S_8_, confirming the formation of high valence Co sites (Figure 4b). The OER free energy diagram on both structures was also calculated (Figure 4c; Figure S23, Supporting Information), which shows that CoO_2_/Co_9_S_8_ tends to follow the adsorption evolution mechanism than the lattice oxygen mechanism. The potential barrier of the potential determining step (PDS) on CoO_2_/Co_9_S_8_ is calculated to be 0.36 eV with the O—O coupling as the PDS,^[^ ^50^, ^51^, ^52^ ^]^ which is lower than that of CoOOH/Co_9_S_8_ (0.98 eV, with OH* deprotonation as the PDS), coincided with the MOR results. Based on the above results, the OER process of CoP/Fe‐Co_9_S_8_ can be schematically described in Figure 4d. First, as a pre‐catalyst, after OER activation, irreversible structural reconstruction of metal oxide formation occurred on the surface of cobalt phosphide. Then, Co^3+^ species (CoOOH) are oxidized to form Co^4+^ species under OER conditions.^[^ ^12^ ^]^ Then, the resulting CoO_2_/Fe‐Co_9_S_8_ undergoes a four‐proton coupling reaction to release O_2_. Due to the low stability of CoO_2_, CoO_2_/Co_9_S_8_ will regenerate into the initial CoOOH/Co_9_S_8_ at a low potential.

### 2.5 Overall Water Splitting in the Electrolytic Cell

The HER performance of the CoP/Fe‐Co_9_S_8_ pre‐catalyst was further evaluated to figure out the ability of overall water splitting. The CoP/Fe‐Co_9_S_8_ also exhibits a low HER overpotential with 62 mV at 10 mA cm^−2^, close to the state‐of‐the‐art Pt/C noble‐metal catalyst (28 mV). It is proved that the catalyst can also act as a highly efficient HER electrocatalyst (details in Supporting Information Figures S24–S29; Table S3, Supporting Information).

Since the CoP/Fe‐Co_9_S_8_ pre‐catalyst showed bifunctional properties of both OER and HER, we evaluated the overall water‐splitting performance of this catalyst in both an alkaline electrolytic cell and an MEA set‐up. In the two‐electrode electrolytic cell equipped with CoP/Fe‐Co_9_S_8_ electrodes, the cell voltage required only 1.44 V at 10 mA cm^−2^, much lower than the reported Co‐based and Fe‐based electrocatalysts (Figure S30; Table S5, Supporting Information). The cell voltage of the CoP/Fe‐Co_9_S_8_ǁCoP/Fe‐Co_9_S_8_ electrocatalytic cell remained stable after 30 h at a current density of 100 mA cm^−2^. The Faraday efficiency of CoP/Fe‐Co_9_S_8_ electrocatalyst to produce H_2_ and O_2_ was 98.85% and 98.92%, respectively (Figure S31, Supporting Information), which further demonstrates the superior performance of the overall water splitting for CoP/Fe‐Co_9_S_8_.

**Figure 5:**
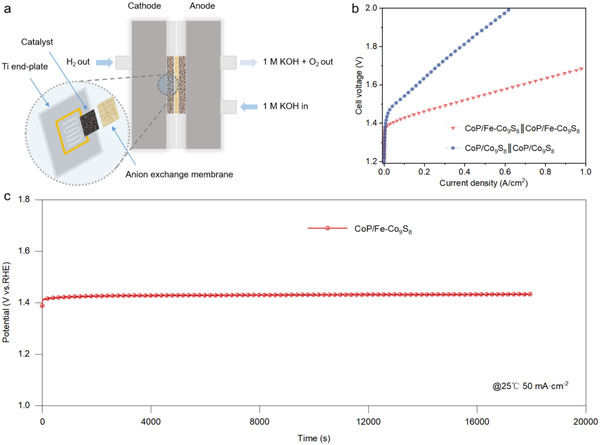
The MEA water electrolysis performance at 25 °C. a) The schematic image of the MEA electrolyzer. b) Polarization curves of MEA electrolyzer in 1 m KOH at a scan rate of 5 mV s^−1^. c) Stability of CoP/Fe‐Co_9_S_8_ electrode in MEA water electrolysis device at 50 mA cm^−2^.

To further verify the application potential, we assembled an MEA electrolyzer using CoP/Fe‐Co_9_S_8_ as both anode and cathode catalyst (**Figure** **5**a). Benefiting from the zero‐gap architecture and the highly efficient catalysts, this MEA electrolyzer only needed 1.68 V to reach 1 A cm^−2^, achieving low‐cost and high‐efficiency overall water splitting (Figure 5b). The MEA electrolyzer and the CoP/Fe‐Co_9_S_8_ catalyst also demonstrated good persistency, and no significant degradation was observed during the 18 000 s test (Figure 5c). Compared with the reported MEA, the MEA equipped with CoP/Fe‐Co_9_S_8_ electrodes shows improved overall water splitting performance (Table S6, Supporting Information). It is proven that the CoP/Fe‐Co_9_S_8_ is a promising catalyst for overall water splitting.

## 3 Conclusion

In summary, in this work, we successfully synthesized CoP/Fe‐Co_9_S_8_ heterostructures using a two‐step hydrothermal method. The catalyst can serve as both an efficient pre‐catalyst for OER and an active catalyst for HER in the alkaline electrolyte, which obtains an ultra‐low overpotential of 156 mV for OER and 62 mV for HER at 10 mA cm^−2^. Electrochemical analysis, ICP‐OES, in situ Raman spectroscopy, and DFT calculations verify that the introduction of Fe could promote the surface reconstruction of the catalyst and form highly active Co(IV)–O sites. Meanwhile, the combination of Fe and S could lead to self‐termination of surface reconstruction, which inhibits the loss of P and S, and forms a stable high valence Co active structure, eventually leading to a highly active and stable OER catalyst. We further assembled an MEA alkaline water electrolyzer using this catalyst as both the anodic and cathodic catalyst. The MEA cell only needs 1.68 V to drive 1A cm^−2^ electrolysis current density, achieving a low‐cost and high‐efficiency overall water splitting device. This work provides new insight into the development of highly active and stable water‐splitting catalysts.

## 4 Experimental Section

See the details in the Supporting Information.

## Conflict of Interest

The authors declare no conflict of interest.

## Supporting information

Supporting Information

## Data Availability

The data that support the findings of this study are available in the supplementary material of this article.
