# Supplementary material for: CoP/Fe‐Co9S8 for Highly Efficient Overall Water Splitting with Surface Reconstruction and Self‐Termination
Source: Adv Sci (Weinh). 2022 Oct 21;9(34):2204742. doi: 10.1002/advs.202204742 (PMC9731716; doi:10.1002/advs.202204742)
Supplement: Supplementary file 1 — Supporting Information [file ADVS-9-2204742-s001.pdf]

ADVANCED SCIENCE
Open Access

Supporting Information

for *Adv. Sci.*, DOI 10.1002/advs.202204742

CoP/Fe-Co_9S_8 for Highly Efficient Overall Water Splitting with Surface Reconstruction and Self-Termination

Xinhong Chen, Yumeng Cheng, Yunzhou Wen, Yaya Wang, Xiao Yan, Jun Wei, Sisi He and Jia Zhou**

Supporting Information

CoP/Fe-Co_9S_8 for highly efficient overall water splitting with surface reconstruction and self-termination

Xinhong Chen[a,b], *Yumeng Cheng*[a], *Yunzhou Wen*[a], *Yaya Wang*[a], *Xiao Yan*[c], *Jun Wei*[d], *Sisi He*[a.b]*, *Jia Zhou*[a,b]*

[a]*State Key Lab of Urban Water Resource and Environment, School of Science, Harbin Institute of Technology Shenzhen, Shenzhen 518055, PR China*
[b]*School of Chemistry and Chemical Engineering, Harbin Institute of Technology, Harbin 150001, PR China.*
[c]*Shenzhen institute of information technology, Shenzhen 518172, PR China*
[d]*Shenzhen Key Laboratory of Flexible Printed Electronics Technology Center, School of Materials Science and Engineering, Harbin Institute of Technology, Shenzhen 518055, PR China*

*Correspondence and requests for materials should be addressed to Sisi He (hesisi@hit.edu.cn) (S.H.) and Jia Zhou (jiazhou@hit.edu.cn) (J.Z.).

Methods

Material and reagents: The nickel foam (NF) (0.58 g·cm^{-2}) was purchased from Kunshan Dessco Electronics Co. Ltd. (Kunshan, China). The reagents acetone (C_3H_6O, 99.5%), hydrochloric acid (HCl, 38.0%), ethanol (C_2H_5OH, 99.7%), Sodium chloride (NaCl, 99.5%), citric acid ($C_6H_8O_7·H_2O$, 99.5%), cobalt sulfate ($CoSO_4·7H_2O$, 99.5%), sodium hypophosphite ($NaH_2PO_2·H_2O$, 99.0%), cobalt nitrate ($Co(NO_3)_2·6H_2O$), iron(III) sulfate hydrate ($Fe_2(SO_4)_3·xH_2O$), thiourea (CH_4N_2S), urea (CH_4N_2O), ammonium fluoride (NH_4F, 99.5%), potassium hydroxide (KOH, 90%) and commercial Pt/C (20 wt%) were purchased from Shanghai Macklin Biochemical Co., Ltd. All reagents were of analytical purity and used without further purification.

Synthesis of CoP/NF: The CoP/NF electrodes were synthesized by a typical hydrothermal method. Before conducting experiments, the NF (0.6×0.6 cm^2) was sequentially cleaned with acetone, ethanol and aqueous HCl solution (3 M) for 30 min to remove residual oil contamination and the surface oxides using an ultrasonic bath. The cleaned NF was immersed into a 10 mL aqueous solution with nitric acid containing 0.02 g $CoSO_4·7H_2O$, 0.1 g $NaH_2PO_2·H_2O$, 0.2 g NH_4F and 0.2 g urea. The aqueous solution with the NF was transferred to a 50 mL Teflon-lined stainless-steel autoclave and heated at 200°C for 12 h. After cooling to room temperature, the NF with precursor was washed with deionized water several times and dried at 60°C.

Synthesis of CoP/Fe-Co_9S_8: The CoP/Fe-Co_9S_8 electrodes were also synthesized by a typical hydrothermal method. The CoP/NF precursor was immersed into a 10 mL aqueous solution containing 0.02 g $Co(NO_3)_2·6H_2O$ and $Fe_2(SO_4)_3·xH_2O$, 0.02 g thiourea, and 0.12 g urea. The aqueous solution with the NF was transferred to a 50 mL Teflon-lined stainless-steel autoclave and heated at 180°C for 12 h. After cooling to room temperature, the NF with precursor was washed with deionized water several times and dried at 60°C.

Synthesis of Fe-Co_9S_8: The synthetic method is the same as that for *CoP/Fe-Co_9S_8* as described except that the CoP/NF precursor is replaced with clean NF.

Synthesis of CoP/Co_9S_8: The synthetic method is the same as that for *CoP/Fe-Co_9S_8* as described except without $Fe_2(SO_4)_3·xH_2O$.

Preparation of Pt/C-NF electrode: Pt/C (2.7 mg) was dispersed into 2 mL mixed solution containing 0.8 mL water, 0.12 mL 5% Nafion solution, and 1.08 mL ethanol. The solution was then ultrasonically treated for 30 minutes to form a uniform catalyst ink (catalyst: 1.35 $mg \cdot mL^{-1}$). 0.1 mL catalyst ink was loaded on the NF electrode (surface area: 0.36 cm^2) repeatedly for 10 times. Consequently, the loading mass of Pt/C-NF was around 1.35 $mg \cdot cm^{-2}$.

Electrochemical Measurements: All the electrochemical experiments were carried out using a conventional three-electrode system on an electrochemical workstation (CS310, Wuhan Kesite Instrument Co., Ltd.) and the prepared samples were used as working electrodes, Hg/HgO soaked in 1 M KOH solution was used as the reference electrode and a graphite rod as the counter electrode. Linear sweep voltammetry (LSV) polarization curves were obtained by the potential dynamic method at the scanning rate of 5 $mV \cdot s^{-1}$ to evaluate the hydrogen evolution (HER), oxygen evolution (OER) and overall water splitting activity of the catalyst in 1 M KOH solutions (pH=13.7). According to E (*vs.* RHE) = E (Hg/HgO) + 0.098 + 0.059pH, all electrode potentials measured relative to the reference potential are translated into the potential versus the reversible hydrogen electrode (RHE). Under constant current density, the stability of the studied electrocatalysts was evaluated by time-potential curves. Under amplitude of 10 mV and frequency range from 100 kHz to 1 Hz, the electrochemical impedance spectroscopy (EIS) experiments were carried out at 1.53 V *vs.* RHE. The specific surface area of all the as-prepared samples was compared by conducting cyclic voltammetric sweeps at various scan rates (from 10 to 130$mV \cdot s^{-1}$) to evaluate the double layer capacitance (C_{dl}). On the other hand, the HER catalytic activity of the pre-catalysts and commercial Pt/C (20 wt%) on NF were also measured. Based on the water drainage method, the Faraday efficiency was measured at 25 $mA \cdot cm^{-2}$ for 1.5 h. Specifically, two 50 ml burettes are reversed-put with two same electrodes of CoP/Fe-Co_9S_8. We recorded the data from0 min to 90 min with 10-minute intervals.

Material Characterization: A scanning electron microscope (SEM; S-4800, Hitachi, Japan) equipped with an energy-dispersive X-ray (EDS) system in 15 kV was used to characterize the morphology and microstructures of the samples. High-resolution transmission electron microscopy (HR-TEM; JEM-2100F, Japan) in 200 kV and an X-ray diffractometer (XRD; D/MAX 2500 diffractometer (Rigaku, Japan)) using Cu Kαradiation (λ =0.154178 nm) were used to analyze the phase and composition of the

samples. The surface valence and chemical composition of as-prepared samples were studied by X-ray photoelectron spectroscopy (XPS; Thermo ESCALAB 250) using Al Kα, and the binding energy is calibrated with C 1s (284.9 eV). In-situ electrochemical Raman measurement was performed with a home-designed electrochemical cell and a HORIBA Raman microscope equipped with a 785 nm laser and a 50 × objective lens. The grating parameter was 1200 GR·mm^{-1}, and the laser intensity was 25%. The Raman spectra were collected by Renishaw In Via Qontor Raman at 0.1 V intervals in the potential range of 1.20-1.50 V *vs.* RHE. The dissolution of ions in the OER process was performed on iCAP7400 (Thermo Fisher Scientific) inductively coupled plasma optical emission spectroscopy (ICP-OES).

Density Functional Theory (DFT) Computational Methods**:** The (110) surface of Co_9S_8 was used as the catalytic substrate in the calculation process. We applied a vacuum layer of at least 25 Å in the Z-direction of the slab models to prevent the interaction between the slabs in the vertical direction. All calculations were carried out using density functional theory with dispersion correction D3 (DFT-D3), and the projected augmented wave(PAW) scheme was implemented in the Vienna ab initio simulation software package (VASP).[1-3] For the structural relaxation and energy calculations, the generalized gradient approximation with Perdew-Burke-Ernzerhof (PBE) parameterization was used.[3] The cut-off energy of the plane wave function is 500 eV. The energy converges criteria were set to be 10^{-3} eV. The geometric configurations were optimized with a gamma-center 2×2×1 k-mesh until the convergence tolerance of force on each atom was smaller than 0.03 eV/Å.

Electrochemical HER Catalytic Performance: Besides the superior OER catalytic performance, the electrocatalyst CoP/Fe-Co_9S_8 is also active for alkaline HER, which has also been evaluated by the same three-electrode system. As shown in **Figure S24a** and **Table S3**, the CoP/Fe-Co_9S_8 exhibits a low HER overpotential with 62 mV at 10 mA·cm^{-2}, which is much superior to those of other electrodes. The CoP/Fe-Co_9S_8 displays the smallest Tafel slope of 59.7 mV·dec^{-1}, showing more efficient HER catalytic kinetics in an alkaline medium (**Figure S24b**). The CoP/Fe-Co_9S_8 exhibits the smallest R_{ct} in the EIS spectra, indicating much faster charge transfer during the HER (**Figure S24c** and **Table S4**). The polarization curves (**Figure S24d**) of the electrode before and after 3000 cycles show no evident change, demonstrating the superior durability of the CoP/Fe-Co_9S_8. Finally, The XPS, SEM and TEM images

(**Figure S25** and **Figure S26**) after the long-term stability test are further conducted to detect the structural change, which demonstrates the decent structural stability of the CoP/Fe-Co_9S_8 catalyst with no obvious surface reconstruction. The DFT calculations (**Figure S27**) were carried out to get further insight into the real active site of CoP/Fe-Co_9S_8during the HER process. The free energy of H adsorption on the S site (-0.027 eV) on the crystal plane of Fe-Co_9S_8 for the CoP/Fe-Co_9S is lower than that on other sites, indicating that the S site on the crystal plane of Fe-Co_9S_8 for the CoP/Fe-Co_9S_8 is the real active site during HER process.

To further explore the source of the superior HER performance of the CoP/Fe-Co_9S_8catalyst, the effect of heterostructure construction on the electronic structure has been investigated by high-resolution XPS spectra. The XPS spectrum of the CoP/Fe-Co_9S_8electrocatalyst further confirms the presence of Co, P, Fe and S (**Figure S28**). The Co 2*p*, P 2*p*, Fe 2*p* and S 2*p* spectra for the samples are shown in **Figure S29**. Compared with the high-resolution XPS of Fe-Co_9S_8, the binding energy of all peaks for the Co 2*p*, Fe 2*p* and S 2*p* spectra of CoP/Fe-Co_9S_8shift negatively, indicating that the Fe-Co_9S_8 phase obtains electrons in the sample CoP/Fe-Co_9S_8.[4-6] Compared with the high-resolution XPS of CoP, the binding energy of all peaks for the Co 2*p* and P 2*p* spectra for the CoP/Fe-Co_9S_8 shift positively, indicating that the CoP phase loses electrons in the sample CoP/Fe-Co_9S_8.[7-10] These results indicate that the electrons transfer from the CoP phase to the Fe-Co_9S_8 phase in CoP/Fe-Co_9S_8 electrocatalyst, resulting in the charge rearrangement between the CoP phase and Fe-Co_9S_8 phase, optimizing the electronic structure of the active center, thus boosting HER catalytic performance.

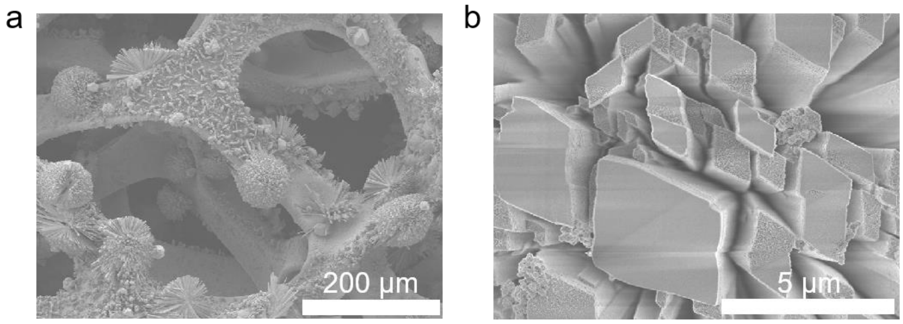

Figure S1. SEM images of the CoP catalyst.

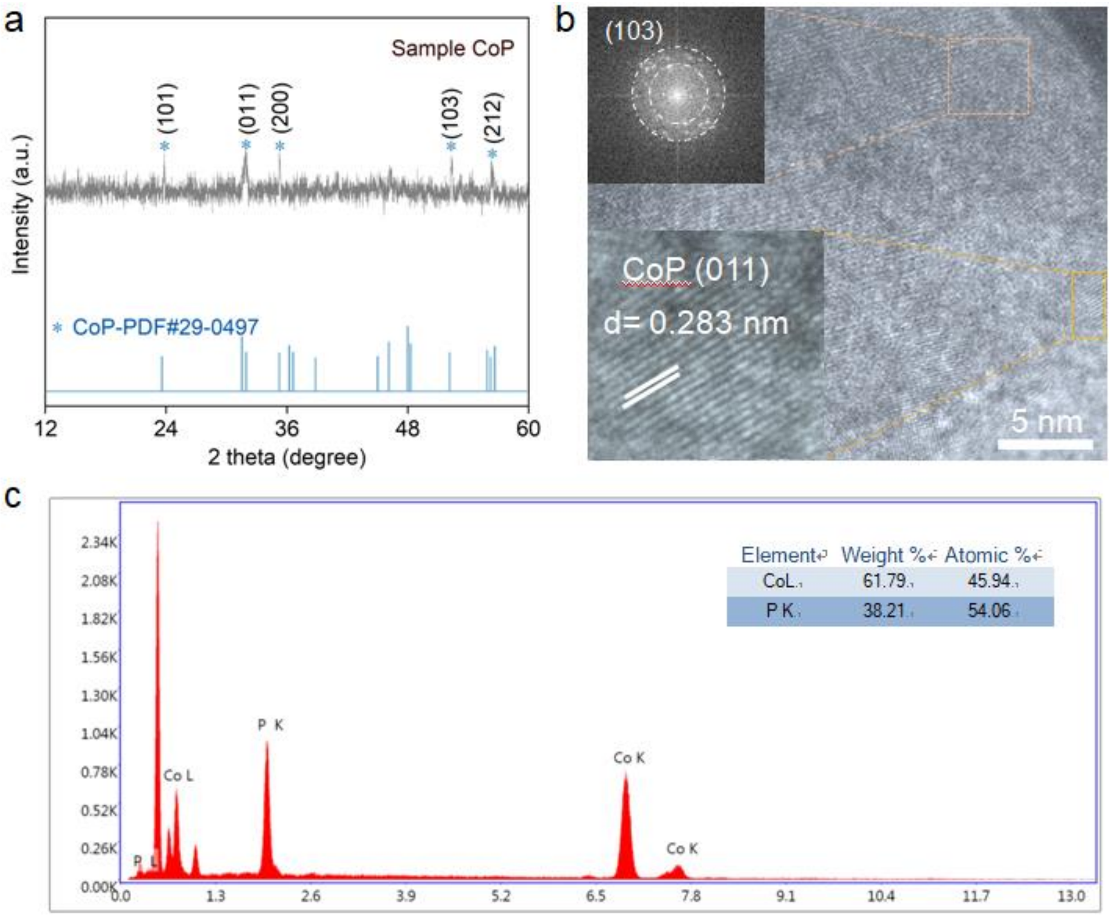

Figure S2. (a) XRD pattern, (b)TEM image and (c) the energy dispersive X-ray (EDS) of the CoP catalyst.

We prepared CoP powder samples and characterized them by XRD, TEM and EDX, as shown in new Figure S2. It is noted that all the XRD diffraction peaks (new Figure S2a) of the CoP sample are respectively consistent with the standard diffraction patterns of CoP (PDF#29-0497). Moreover, the HRTEM image of the CoP sample (new Figure S2b) confirmed obvious crystalline planes. The lattice distances of 0.283 and 0.175 nm unambiguously correspond to the (011) and (103) planes of CoP, respectively. Then, in new Figure S2c, the EDS of the CoP sample indicated that the atomic ratio of Co and P is about 1, which agrees with the composition of CoP. Therefore, the catalyst obtained in the first-step hydrothermal can be considered as the CoP phase.

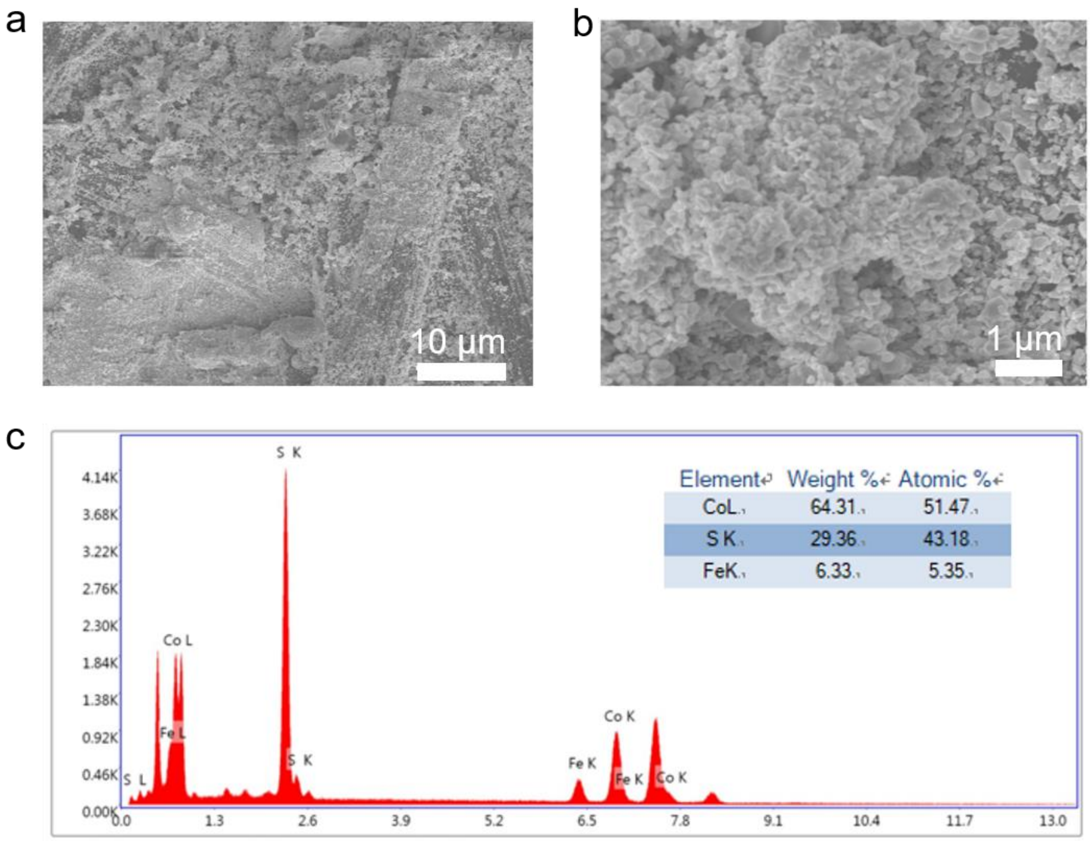

Figure S3. (a, b) SEM images and (c) the EDS of the Fe-Co_9S_8 catalyst.

According to the atomic content of each element in Figure S3, it can be calculated that the atomic ratio of Co, Fe and S in the Fe-Co_9S_8 sample is about 9.62: 1: 8.07.

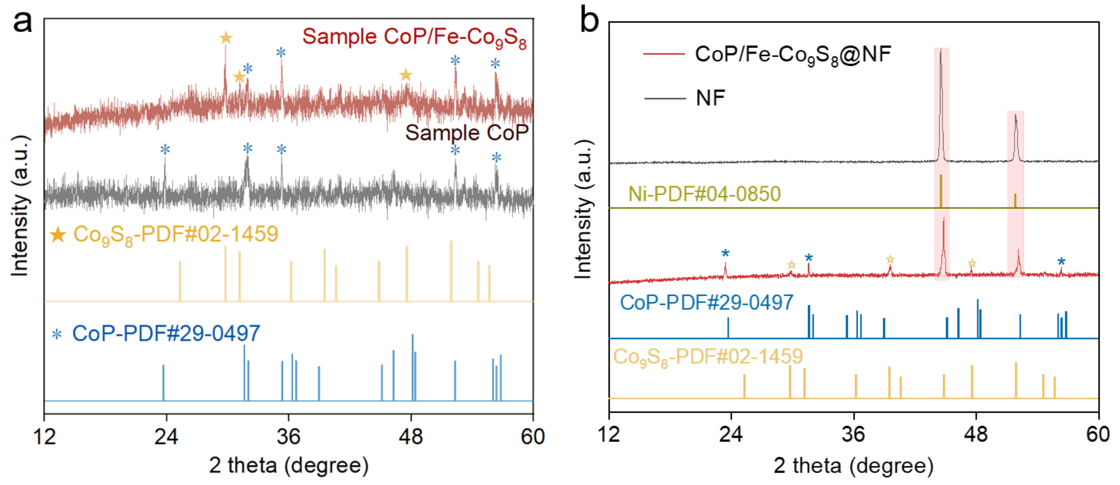

Figure S4. XRD pattern of (a) the CoP/Fe-Co_9S_8powder sample and (b) the CoP/Fe-Co_9S_8 supported on NF.

To further confirm the phase composition of the catalyst CoP/Fe-Co_9S_8, we prepared CoP powder samples and CoP/Fe-Co_9S_8 powder samples and characterized them by XRD (Figure S4). The results are shown in Figure S4, from which it can be observed that CoP/Fe-Co_9S_8 powder is completely composed of the CoP phase and Co_9S_8 phase.

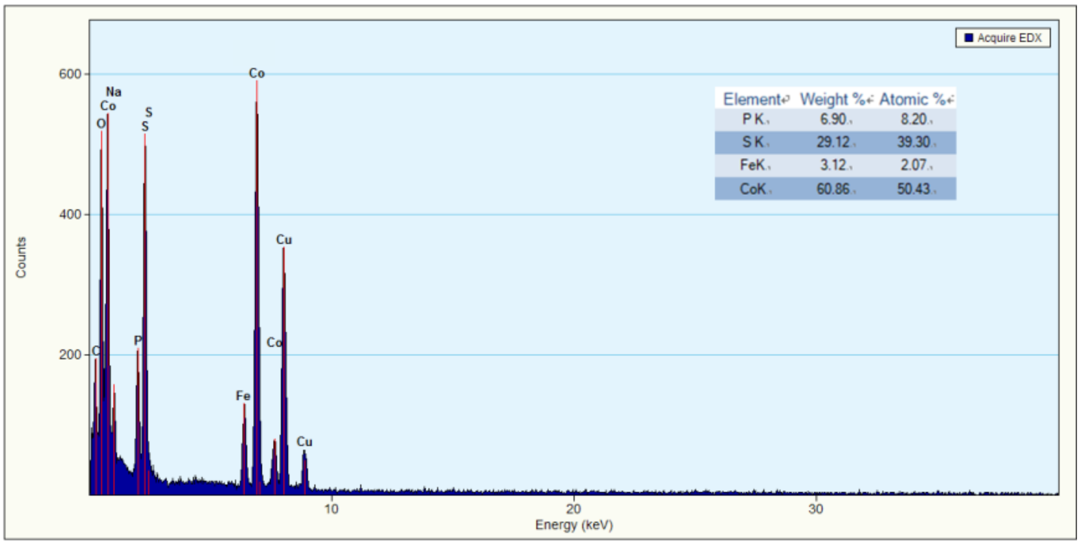

Figure S5. The energy dispersive X-ray (EDX) of the powder sample for the CoP/Fe-Co_9S_8 catalyst.

According to the atomic content of each element in Figure S5, it can be calculated that the atomic ratio of Co, P, Fe and S in the CoP/Fe-Co_9S_8 sample is about 24.96:3.96:1:18.98.

In addition, we prepared CoP/Fe-Co_9S_8 powder by a two-step hydrothermal synthesis method. After drying, the mass of the CoP sample after the first reaction (verified by XRD with new Figure S2) was about 1.8mg. The mass of the final CoP/Fe- Co_9S_8 powder (Co_9S_8 verified by XRD of new Figure S4 and Fe doping verified by XPS and EDX of Figure S15 and new Figure S5) was 8.2 mg. Therefore, it can be calculated that the mass content of CoP and Fe-Co_9S_8 components is about 21.6% and 78.4% respectively.

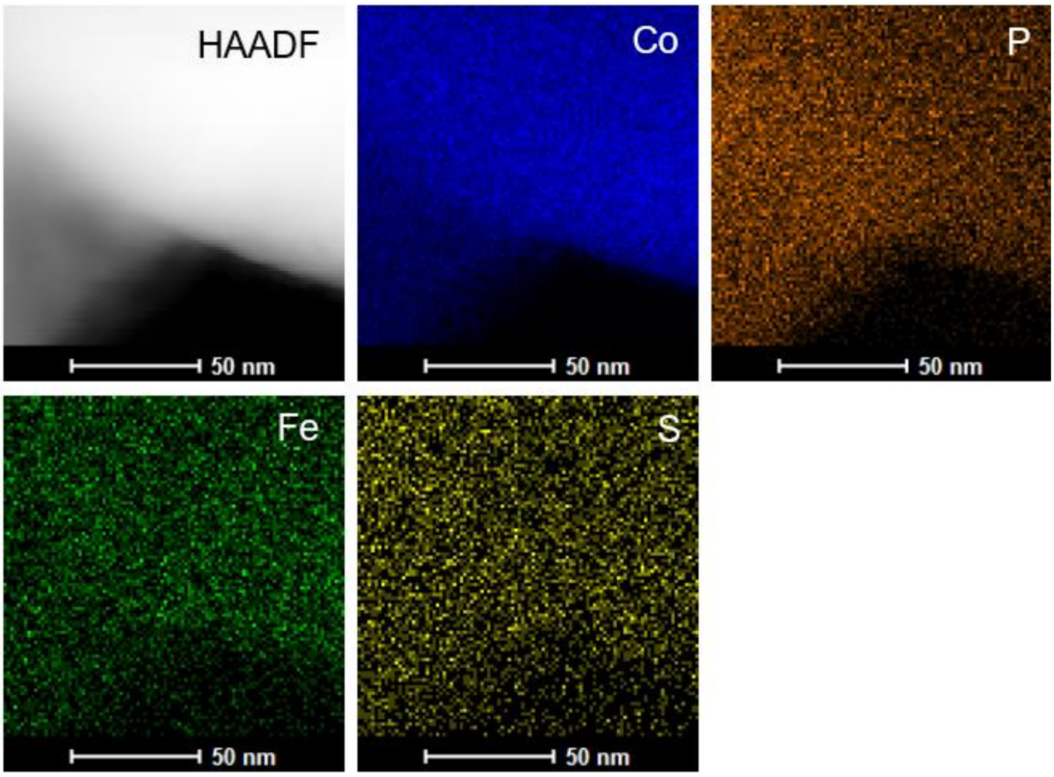

Figure S6. The elemental mapping images of the powder sample for the CoP/Fe-Co_9S_8 catalyst at 50 nm.

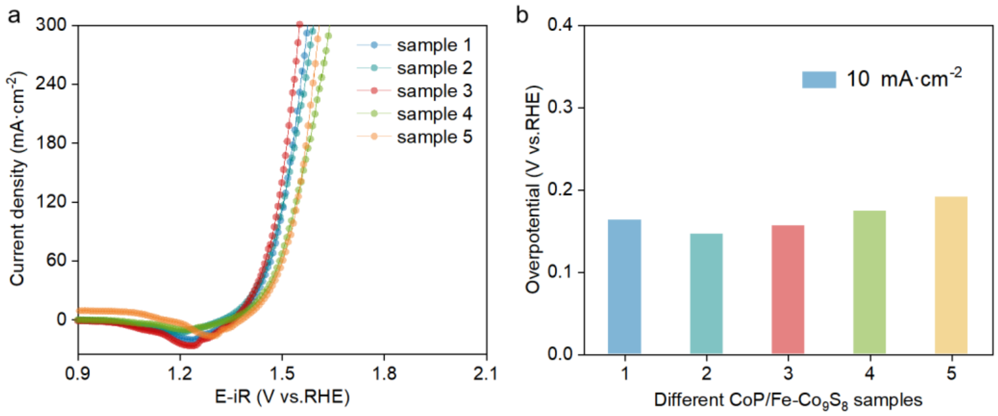

Figure S7. (a) LSV curves for the five independent repetitions of CoP/Fe-Co_9S_8 samples in 1M KOH solution. (b) corresponding overpotential at 10 mA·cm^{-2}.

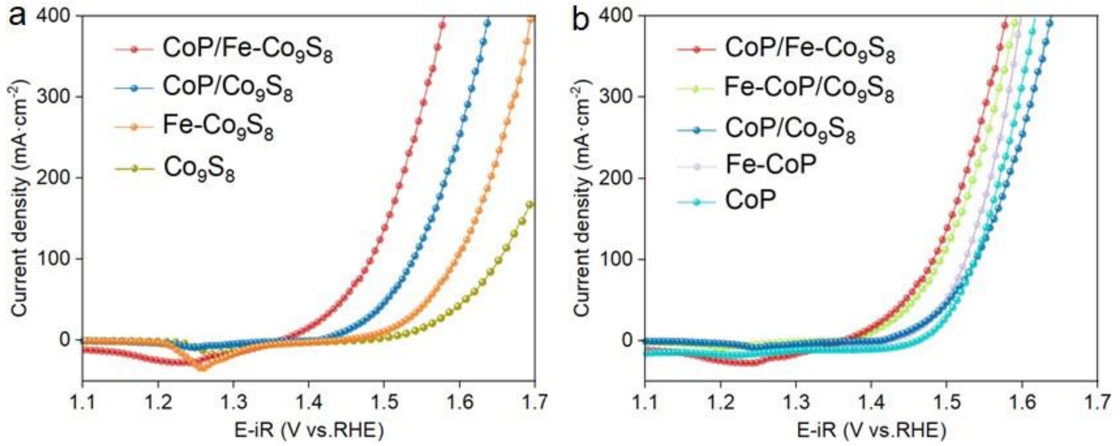

Figure S8. LSV curves of (a) Co_9S_8, (b) Fe-CoP/Co_9S_8 and Fe-CoP samples in 1M KOH solution.

Fe was incorporated with Co_9S_8 rather than CoP. First, we evaluated the catalytic performance of pure Co_9S_8, and further compared it with the performance of Fe-Co_9S_8, CoP/Co_9S_8 and CoP/Fe-Co_9S_8 catalysts. As shown in Figure S8a, the catalytic performance of Fe-Co_9S_8 is improved compared with that of pure Co_9S_8, which indicates that Fe doped in Co_9S_8 is beneficial to enhance catalytic activity. In addition, we also measured the catalytic performance of Fe-CoP and further compared it with the performance of CoP, Fe-CoP/Co_9S_8 and CoP/Fe-Co_9S_8 catalysts. As shown in Figure S8b, the catalytic performance of Fe-CoP is similar to that of CoP, which indicates that Fe doping in CoP has little effect on catalytic activity. The catalytic performance of CoP/Fe-Co_9S_8 is better than that of CoP/Co_9S_8 and Fe-CoP/Co_9S_8, which proves that Fe is doped in Co_9S_8, thus boosting the performance.

Then, the XPS spectra of Fe 2p in Figure S14c indicated the formation of Fe-S chemical bonds, which can prevent the serious loss of P and S. Since the electronegativity of S (2.5) is greater than that of P (2.1), the Fe-P bond formed in Fe doped-CoP is weaker than the Fe-S bond formed in Fe doped-Co_9S_8, leading to the possible loss of more active phase for the Fe-CoP/Co_9S_8. However, the content of Co and S in the ICP-OES test (Figure 3h and i) remained unchanged, indicating that Fe was more incorporated with Co_9S_8 rather than CoP.

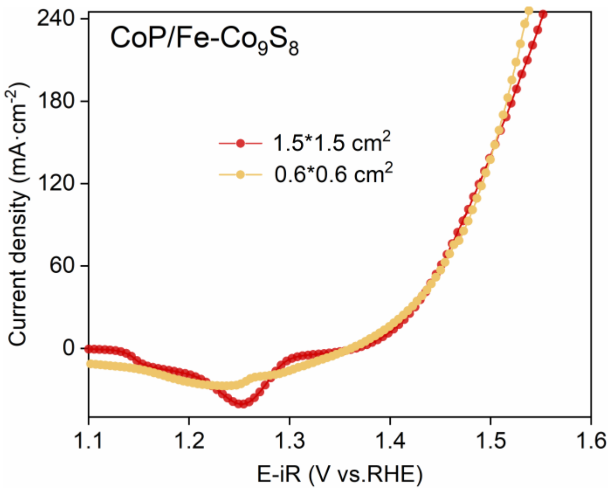

Figure S9. LSV curves of CoP/Fe-Co_9S_8 samples with a larger area of 1.5*1.5 cm^2 in 1M KOH solution.

We carried out the polarization curve of the CoP/Fe-Co_9S_8 with a larger area of 1.5*1.5 cm^2, and the OER overpotential of the catalyst at 10mA·cm^{-2} is 167 mV (1.5*1.5 cm^2) compared with the value of 156 mV with an area of 0.6*0.6 cm^2. The catalyst still exhibited superior catalytic performance with a larger area.

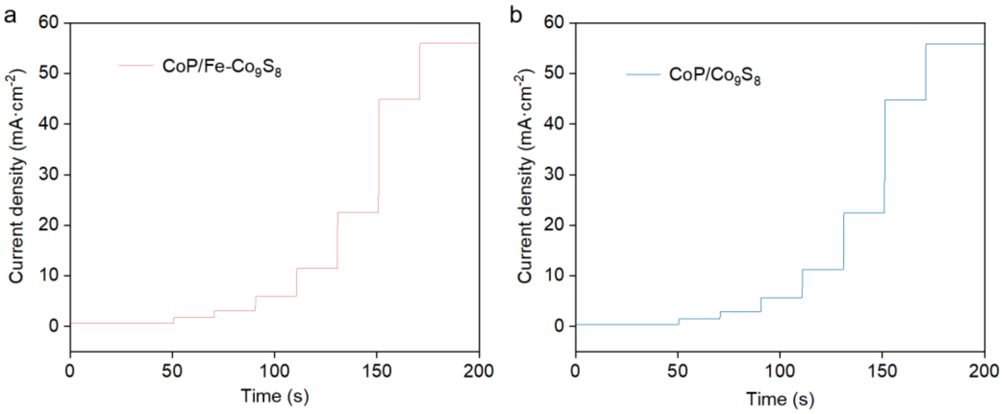

Figure S10. Tafel steady-state tests of (a) $CoP/Fe\text{-}Co_9S_8$ and (b) CoP/Co_9S_8 catalysts.

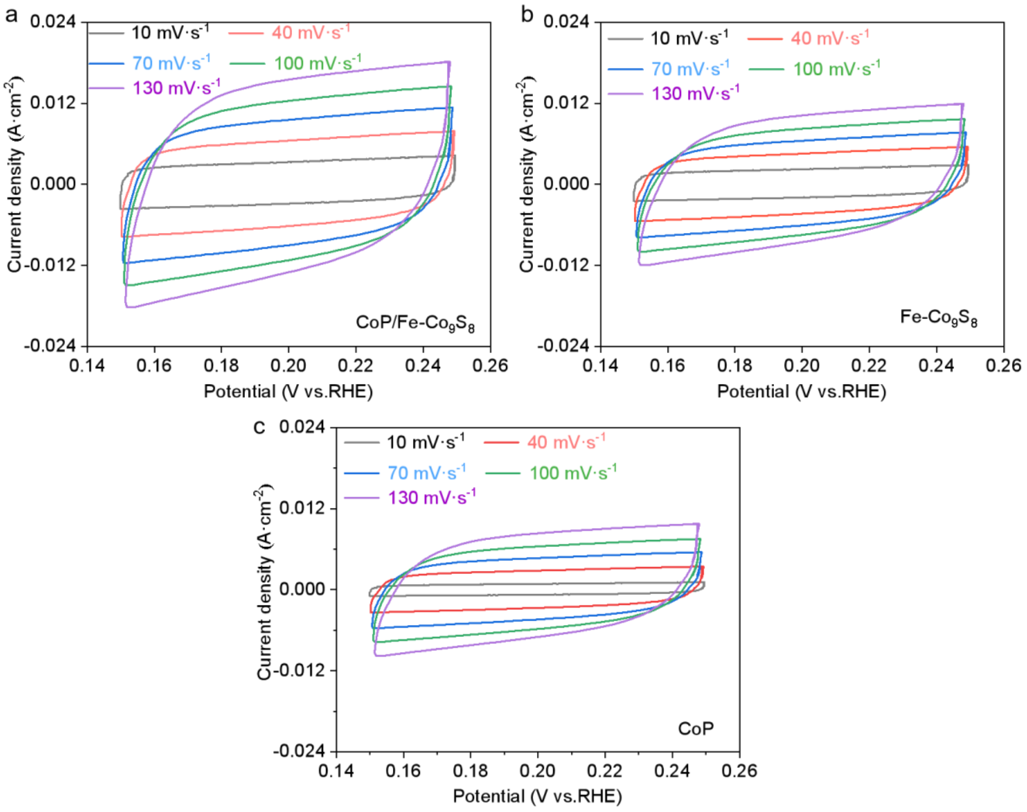

Figure S11. CV curves of (a) CoP/Fe-Co_9S_8, (b) Fe-Co_9S_8 and (c) CoP at scan rates from 10 to 130 mV·s^{-1}.

Since the electrochemical surface area (ECSA) plays an important role in the assessment of activity, we further perform an investigation on its influence. First, the CV curves are recorded in a non-Faradic region (Figure S8). Next, the differences in current density variation ($\Delta j = j_a - j_c$) at the potential of 0.2 V vs RHE plotted against scan rate are fitted to estimate the electrochemical double-layer capacitances (C_{dl}), yielding 113, 73.1 and 70.1 mF·cm^{-2} for CoP/Fe-Co_9S_8, Fe-Co_9S_8 and CoP respectively (Figure 2f). Since the C_{dl} for a flat surface is generally found to be in the range of 20-60 μF·cm^{-2}, the ECSA is calculated by using a factor of 40 μF·cm^{-2}.

$$ECSA_{CoP/Fe\text{-}Co9S8} = \frac{113 mF/cm^2}{40 \mu F/cm^2 \, per cm^2_{ECSA}} = 2825 \quad cm^2_{ECSA}$$

$$ECSA_{Fe\text{-}Co9S8} = \frac{73.1 mF/cm^2}{40 \mu F/cm^2 \, per cm^2_{ECSA}} = 1827.5 \quad cm^2_{ECSA}$$

$$ECSA_{CoP} = \frac{70.1 mF/cm^2}{40 \mu F/cm^2 \, per cm^2_{ECSA}} = 1752.5 \quad cm^2_{ECSA}$$

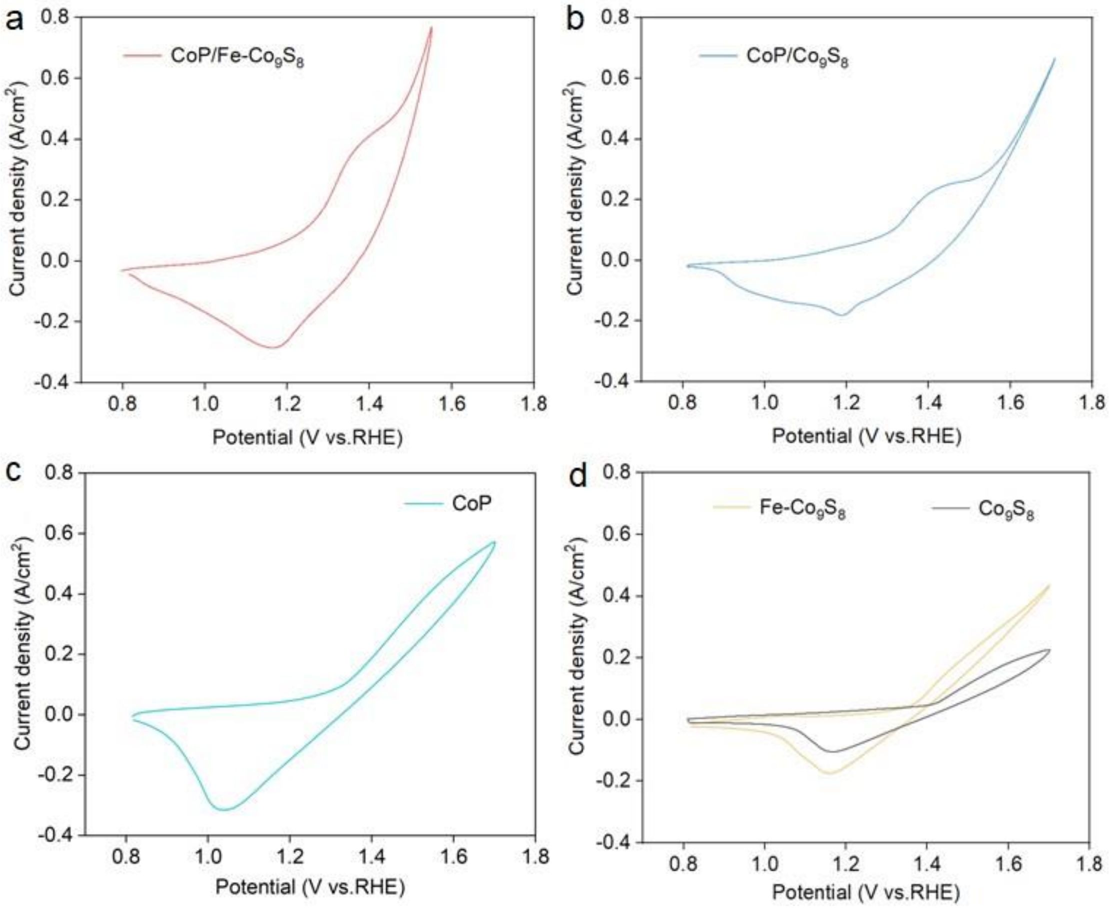

Figure S12. CV curves in the potential range of 0.8–1.7 V versus RHE at 300 mV·s^{-1}. (a) CoP/Fe-Co_9S_8, (b) CoP/Co_9S_8, (c) CoP, (d) Fe-Co_9S_8 and Co_9S_8.

The number of active sites is calculated using the following equation:

$$n_{Co} = \frac{Q_{Co}}{F} \times NA$$

where Q_{Co} is the integration area of Co redox peak from CV curves, F is the Faraday constant, NA is Avogadro's constant, assuming that Co^{2+}/Co^{4+} is a two-electron process.

The Q_{Co} of CoP/Fe-Co_9S_8 (0.55 C), CoP/Co_9S_8 (0.37 C), CoP (0.44 C), Fe-Co_9S_8 (0.20 C) and Co_9S_8 (0.16 C) can be calculated by integrating Co redox peak from CV curves (**Figure S12**). The number of active sites of the catalyst CoP/Fe-Co_9S_8 is the largest, thus showing the best catalytic performance.

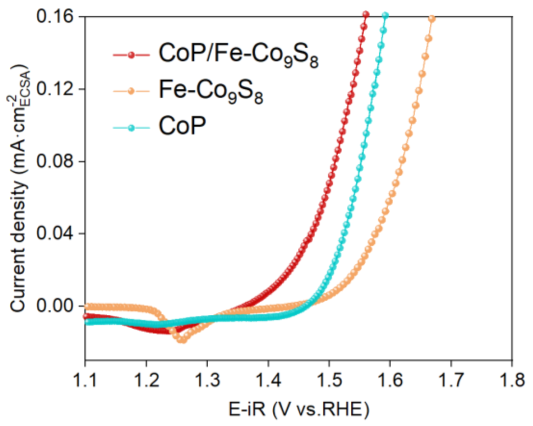

Figure S13. The intrinsic catalytic activity of CoP/Fe-Co_9S_8, Fe-Co_9S_8 and CoP catalyst evaluated by the iR-corrected polarization curves in terms of the current density normalized with ECSA.

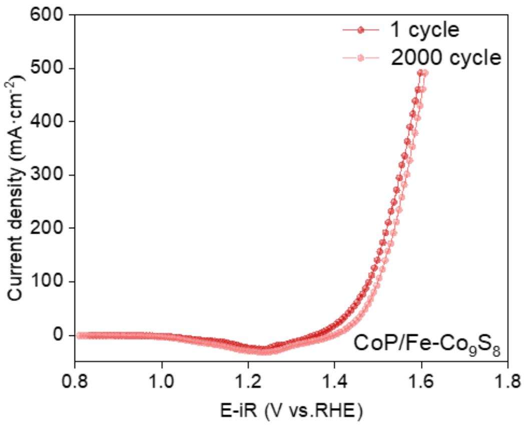

Figure S14. Polarization curves for the CoP/Fe-Co_9S_8 before and after 2000 CV cycles.

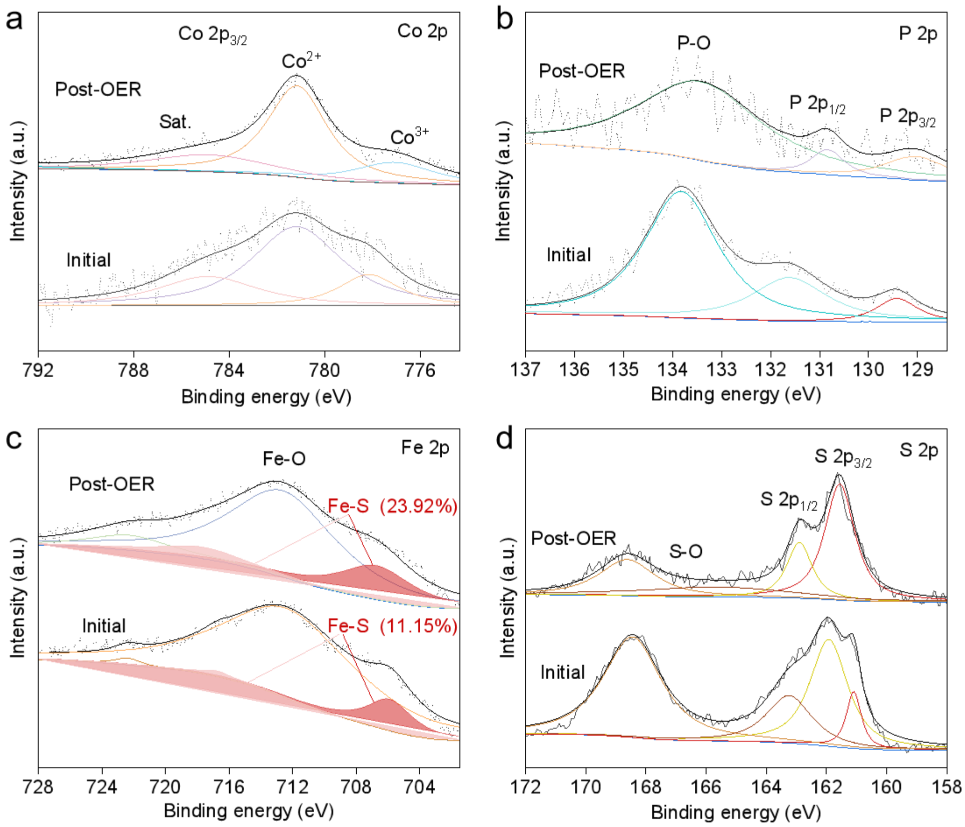

Figure S15. High-resolution XPS spectra of the $CoP/Fe\text{-}Co_9S_8$ undergoing an OER process in 1 M KOH at j_{100} for 40 h, denoted as Post-OER: (a) Co 2*p*, (b) P 2*p*, (c) Fe 2*p* and (d) S 2*p*.

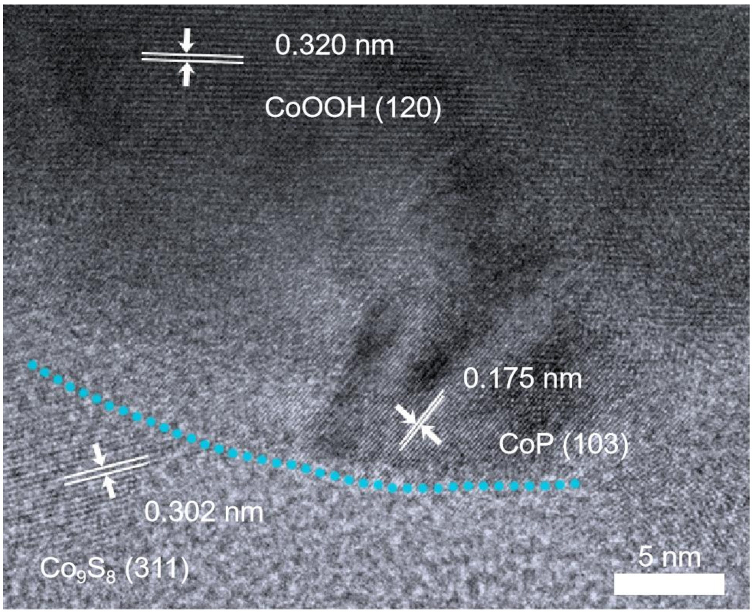

Figure S16.HR-TEM image of CoP/Fe-Co_9S_8 after OER stability test.

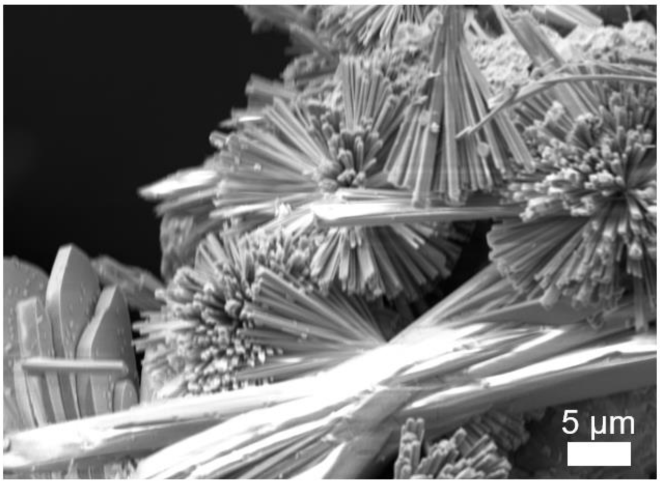

Figure S17. SEM image of $CoP/Fe\text{-}Co_9S_8$ nanorods on NF after OER tests.

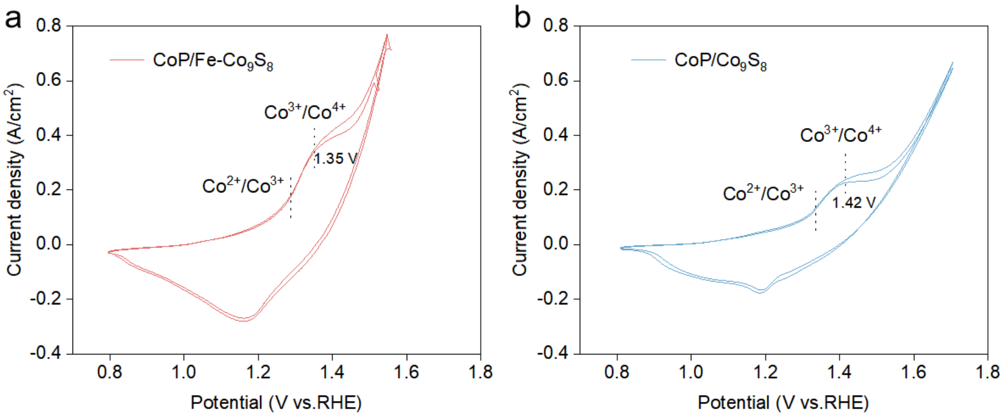

Figure S18. CV curves in the potential range of 0.8–1.7 V versus RHE at 300 mV·s^{-1}. (a) CoP/Fe-Co_9S_8 and (b) CoP/Co_9S_8.

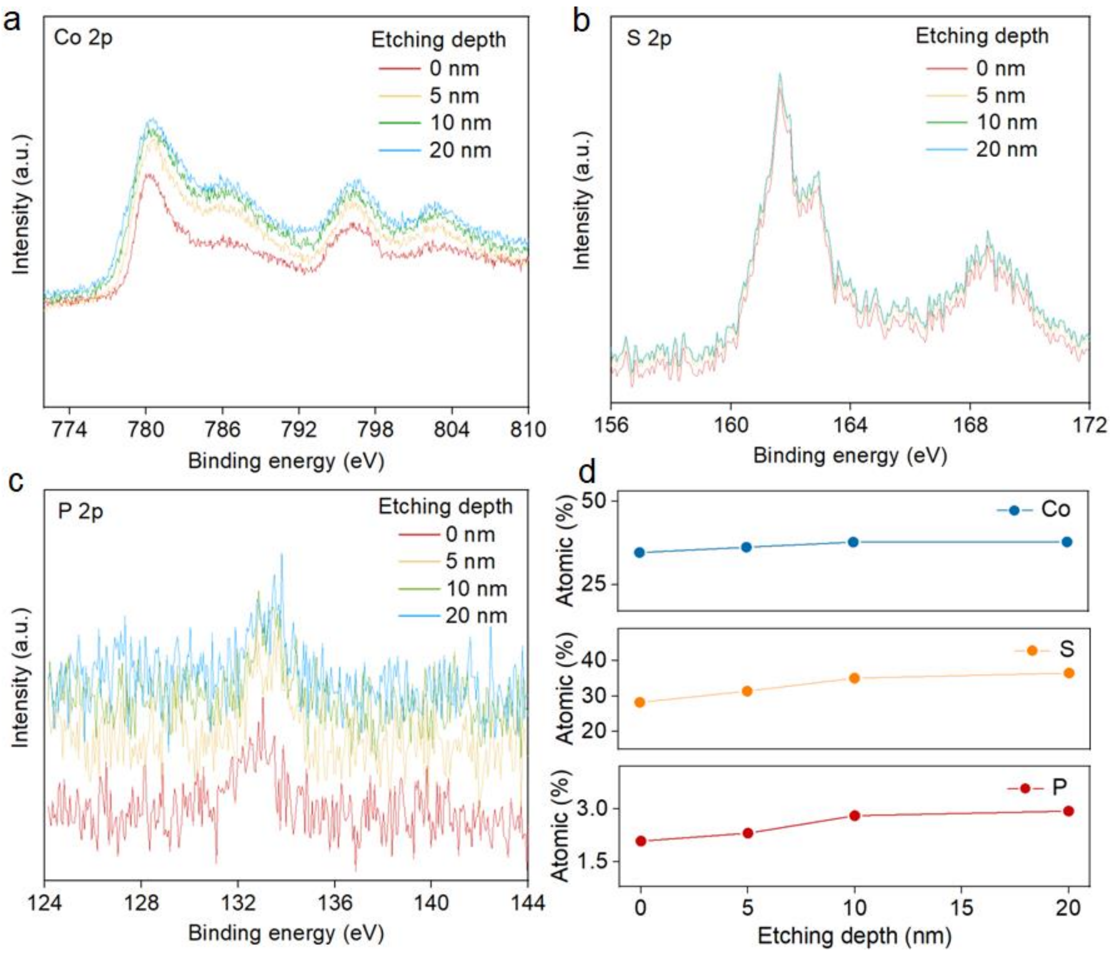

Figure S19. The XPS spectra of (a) Co, (b) P and (c) S for the catalyst CoP/Fe-Co_9S_8 at different etching depths. (d) The variation of atomic content with etching depth.

To quantitively verify the depth of reconstruction, we carried out composition analysis at different depths for the catalyst after 24 h OER reaction using Ar-ion etching XPS depth analysis. As shown in Figure S19, after etching at different depths, the XPS spectra of Co, P and S show that the atomic content changes. When the etching depth reaches 10nm, the element content remains unchanged, indicating that the catalyst is reconstructed only on the surface. According to current reports (Adv. Mater. 2021, 33, 2007344), the reconstructed catalysts display a low degree of surface reconstruction, and the reconstruction thickness is denoted as T_{lsr} (0 <T_{lsr}< 10 nm). Therefore, the reconstruction of catalyst CoP/Fe-Co_9S_8 in this work belongs to surface reconstruction.

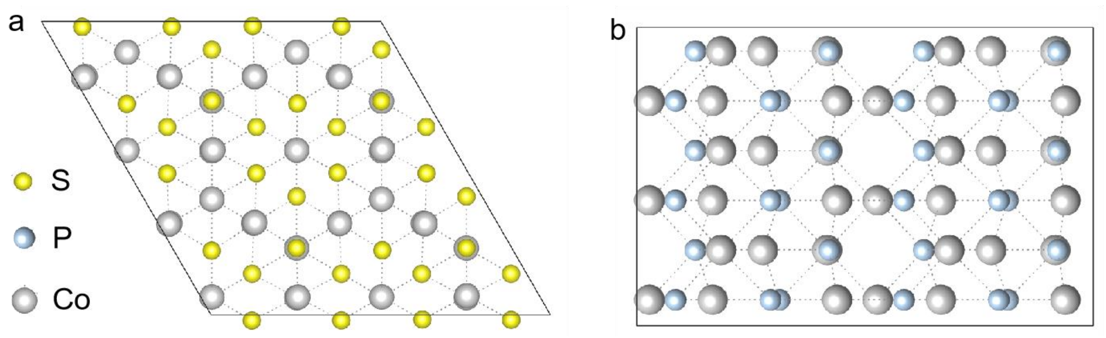

Figure S20. The top view of the DFT calculated crystal plane model: (a) Co_9S_8 (100) and (b) CoP (011).

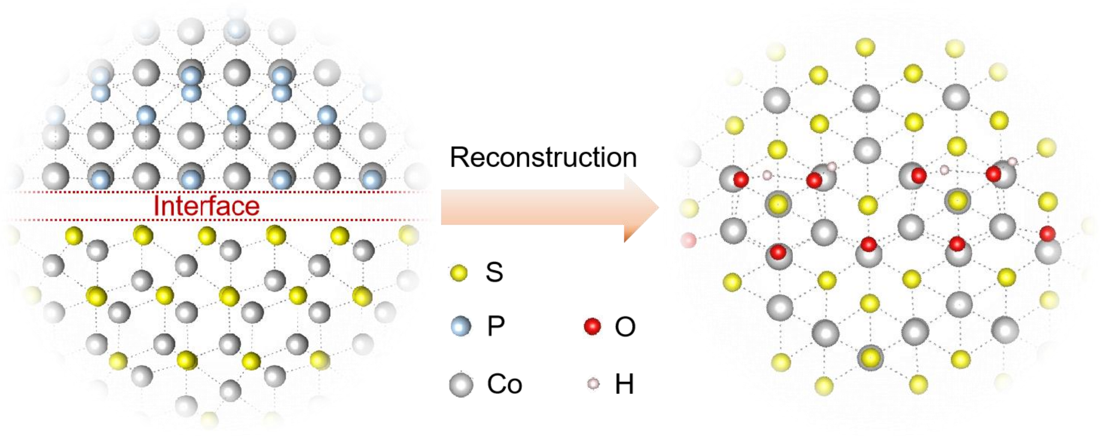

Figure S21. The DFT calculation model for the reconstruction process from CoP/Co_9S_8 to $CoOOH/Co_9S_8$ during catalytic OER.

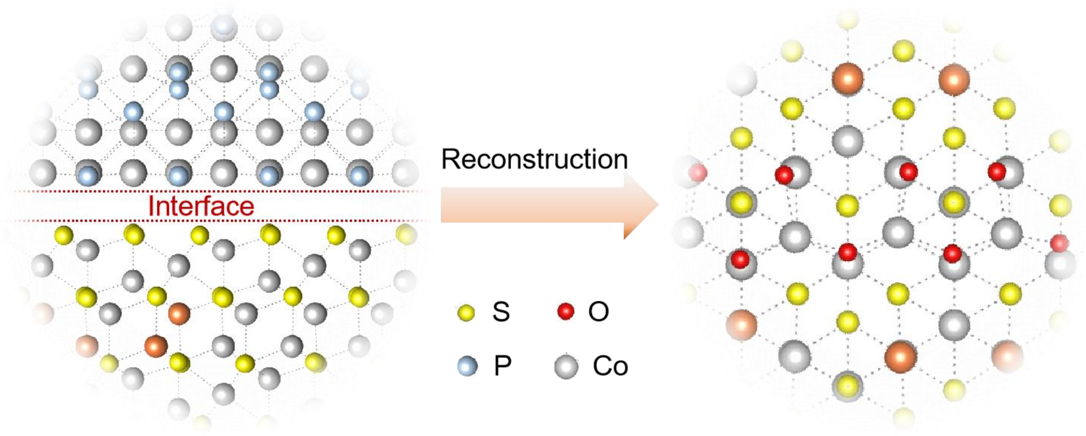

Figure S22. The DFT calculation model for the reconstruction process from CoP/Fe-Co_9S_8 to CoO_2/Fe-Co_9S_8 during catalytic OER.

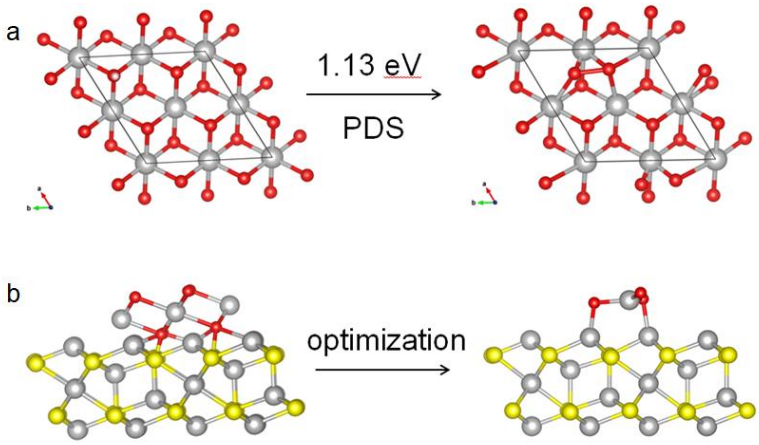

Figure S23. The DFT calculation model for the LOM mechanism of CoP/Fe-Co_9S_8 during catalytic OER.

By calculating the lattice oxygen path (Figure S23a), it was found that the energy barrier of the potential determining step is 1.13 eV, which is higher than the O-O coupling reaction energy (0.36) of CoO_2/Co_9S_8 in this work. Then, the model of CoO_2 cell loading on the surface of Co_9S_8 was further built, as shown in Figure S23b. However, the following calculation found that the model structure of CoO_2/Co_9S_8 would change when the reaction intermediate was adsorbed on the O site, leading to a sharp rise in energy. Therefore, the calculation of the lattice oxygen path of CoO_2/Co_9S_8 also confirmed that Co^{4+} tends to follow the traditional adsorption evolution mechanism in our research.

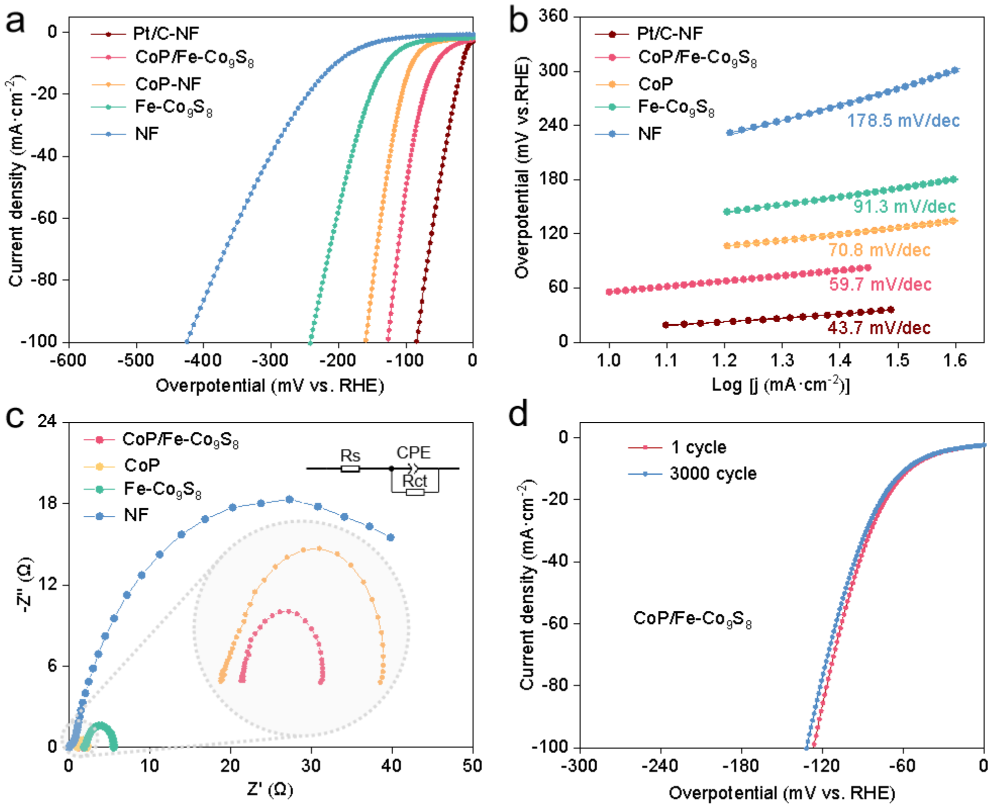

Figure S24. HER catalytic performance of electrocatalyst in 1M KOH alkaline medium. (a) Polarization curves of the CoP/Fe-Co_9S_8 and other catalysts at 5 mV·s^{-1}. (b) Tafel slope. (c) Nyquist plots. (d) Comparison of polarization curves before and after 3000 CV cycles.

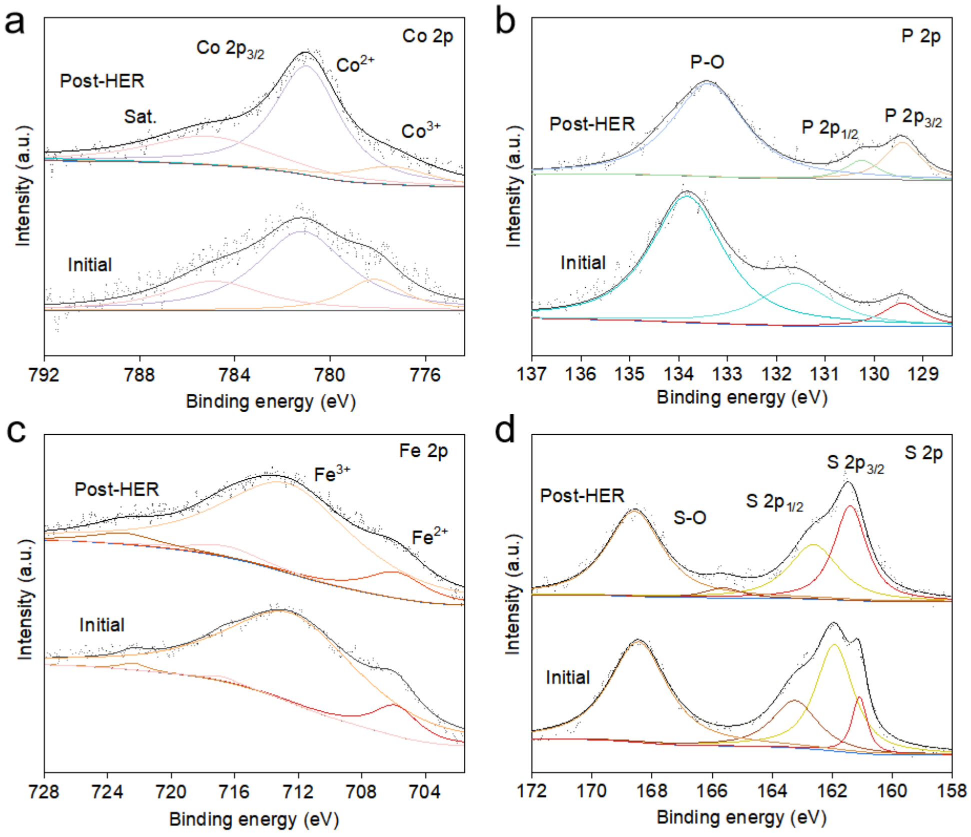

Figure S25. High resolution XPS spectra of the CoP/Fe-Co_9S_8 undergoing a HER process in 1 M KOH after 3000 CV cycles, denoted as Post-HER: (a) Co 2*p*, (b) P 2*p*, (c) Fe 2*p* and (d) S 2*p*.

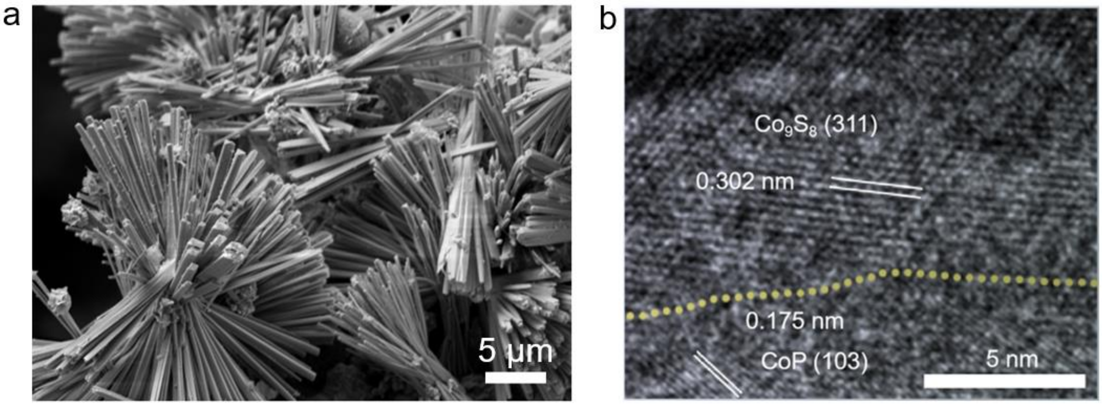

Figure S26. (a) SEM image of CoP/Fe-Co_9S_8 after HER stability test. (b) HR-TEM image of CoP/Fe-Co_9S_8 nanorods on NF after HER tests.

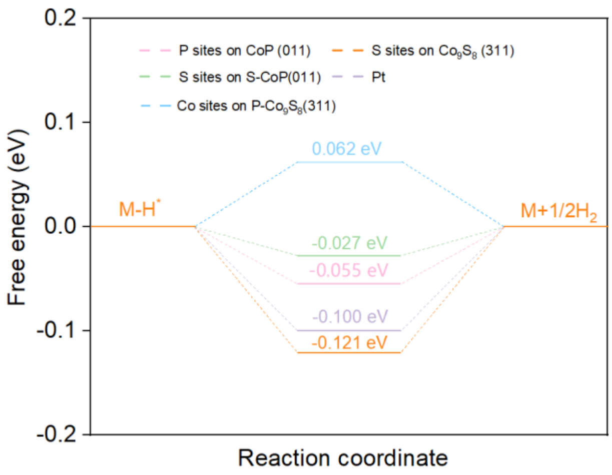

Figure S27. The free energy of H adsorption on the various absorb site for the CoP/Fe-Co_9S_8.

The DFT calculations (Figure S27) were carried out to get further insight into the real active site of CoP/Fe-Co_9S_8 during the HER process. We calculated the adsorption free energy of H intermediate at Co, P sites on the crystal plane of CoP for the CoP/Fe-Co_9S_8 and Co, Fe, S sites on the crystal plane of Fe-Co_9S_8 for the CoP/Fe-Co_9S_8 respectively, and the results are shown in the figure below. It can be concluded that the free energy of H adsorption on the S site (-0.027 eV) on the crystal plane of Fe-Co_9S_8 for the CoP/Fe-Co_9S_8 is lower than that on other sites, indicating that the S site on the crystal plane of Fe-Co_9S_8 for the CoP/Fe-Co_9S_8 is the real active site during HER process.

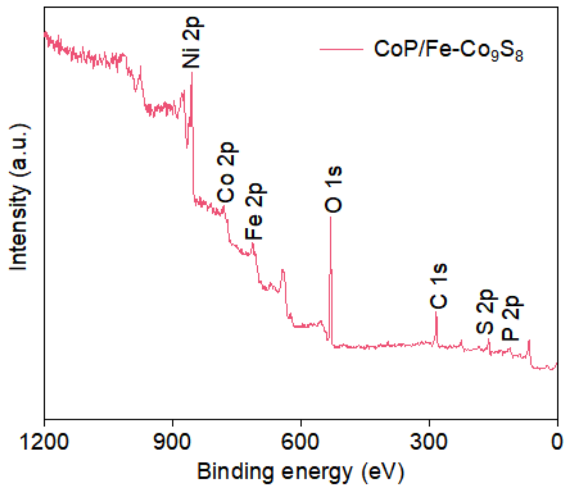

Figure S28. The full XPS scan spectrum of the CoP/Fe-Co_9S_8.

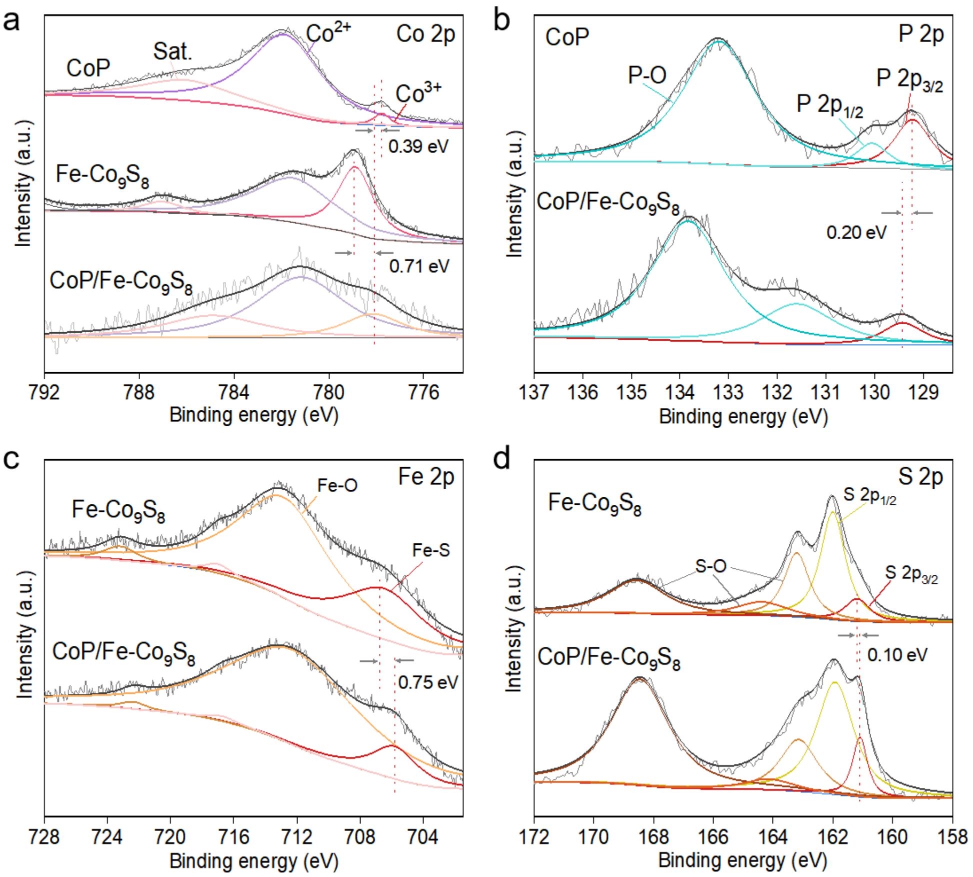

Figure S29. High-resolution XPS spectra (a) Co 2*p*; (b) P 2*p*; (c) Fe 2*p*; (d) S 2*p* of CoP/Fe-Co_9S_8, Fe-Co_9S_8 and CoP.

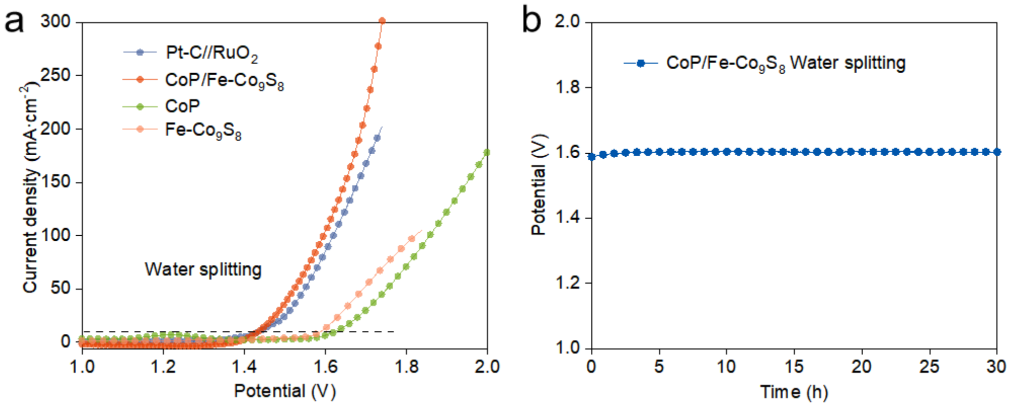

Figure S30. Overall water splitting performance measured in 1 M KOH solution at 25℃. (a) Polarization curves of the CoP/Fe-Co_9S_8 bifunctional catalysts at 5 $mV \cdot s^{-1}$ in a two-electrode electrolyzer. (b) Durability tests of the electrolyzer under the constant current densities of 100 $mA \cdot cm^{-2}$.

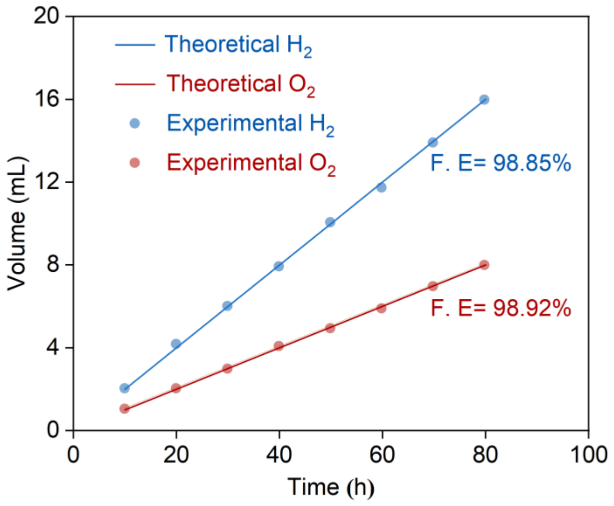

Figure S31. Faradic efficiency of the CoP/Fe-Co_9S_8‖CoP/Fe-Co_9S_8 electrocatalytic cell for overall water splitting.

Table S1. The OER performances of the as-prepared CoP/Fe-Co_9S_8 electrode and other electrodes with Co-based and Fe-based electrocatalysts in 1.0 M KOH.

Catalysts	j (mA/cm^2)	η (mV)	Reference
CoP/Fe-Co_9S_8	10	156	**This work**
CoP/Co_9S_8	10	214	This work
Fe-Co_9S_8	10	266	This work
CoP	10	249	This work
FeNi-LDH/CoP	10	231	Angew. Chem. Int. Ed. Engl. 2019, 58, 11903-11909.
LiCoBPO/NF	10	293	Energy Environ. Sci. 2019, 12, 988-999
MoS_2/Co_9S_8/Ni_3S_2/Ni	10	166	J. Am. Chem. Soc. 2019, 141, 10417-10430.
Fe_3O_4/FeS_2	10	253	J. Mater. Chem. A 2020, 8, 14145-14151.
NiCoMo	10	304	ACS Energy Lett. 2019, 4, 952-959.
F-Co_2P/NF	10	307	J. Mater. Chem. A 2021, 9, 22626-22634
$CoNiMoO_4$-21/CuO_x/CF	10	221	Adv. Energy Mater. 2021, 2102361
V-CoP	10	267	Adv. Energy Mater. 2021, 2101758
$NiCo_2S_4$-4	10	243	Adv. Funct. Mater. 2019, 29, 1807031.
FeP/Ni_2P	10	154	Nat. Commun. 2018, 9, 2551.
NiFeP-NS-O	10	100	Appl. Catal. B Environ. 2022, 302, 120862
Ru_2O	10	387	J. Mater. Chem. A. 2016, 4, 3068-3076.

Table S2. Geometric values of the electronic elements extracted from the electrical equivalent circuit model in Fig. 2e.

	Rs	Rct	CPE-T	CPE-P
$CoP/Fe\text{-}Co_9S_8$	0.656	0.624	1.464	0.704
CoP/Co_9S_8	0.634	1.307	0.803	0.813
$Fe\text{-}Co_9S_8$	0.622	3.223	0.198	0.806
CoP	0.583	0.938	1.225	0.835

Rs related to the series resistance. Rct denotes the charge transfer resistance. CPE is the constant phase angle element, which represents the double layer capacitance.

Table S3. The HER performances of the as-prepared CoP/Fe-Co_9S_8 electrode and other electrodes with Co-based and Fe-based electrocatalysts in 1.0 M KOH.

Catalysts	j (mA/cm^2)	η (mV)	Reference
CoP/Fe-Co_9S_8	10	62	**This work**
Fe-Co_9S_8	10	129	This work
CoP	10	91	This work
Co_3S_4/EC-MOF	10	84	Adv. Mater. 2019, 31, 1806672.
$NiCo_2S_4$-4	10	80	Adv. Funct. Mater. 2019, 29, 1807031.
$MoS_2/Co_9S_8/Ni_3S_2$/Ni	10	113	J. Am. Chem. Soc. 2019, 141, 10417-10430.
FeS_2/CoS_2	10	78.2	Small 2018, 14, 1801070.
δ-FeOOH NSs/NF	10	108	Adv. Mater. 2018, 30, 1803144.
NiCoMo	10	52	ACS Energy Lett. 2019, 4, 952-959.
$Fe_{17.5\%}$-Ni_3S_2/NF	10	47	ACS Catal. 2018, 8, 5431.
$CoNiMoO_4$-21/CuO_x/ CF	10	46	Adv. Energy Mater. 2021, 2102361
V-CoP	10	46	Adv. Energy Mater. 2021, 2101758
Fe-CoP/Ti	10	78	Adv. Mater. 2017, 29, 1602441.
FeB_2	10	61	Adv. Energy Mater. 2017, 7, 1700513.
Co_2P/CoNPC	10	208	Adv. Mater. 2020, 32, 2003649.
B-CoP/CNT	10	61	Angew. Chem. Int. Ed. Engl. 2020, 59, 4154-4160
$Fe_{0.29}Co_{0.71}P$-LDH/NF	10	74	Nano Energy 2020, 67, 104174
Pt/C/CC	10	20	Adv. Energy Mater. 2018, 1800935.
Pt/CC	10	28	ACS Catal. 2019, 9, 3744-3752
Pt/C	10	40	Adv. Energy Mater. 2020, 10, 1903854

Table S4. Geometric values of the electronic elements extracted from the electrical equivalent circuit model in Fig. S18c.

	Rs	Rct	CPE-T	CPE-P
CoP/Fe-Co_9S_8	1.591	0.3	0.061	0.952
Fe-Co_9S_8	1.104	1.4	0.030	0.910
CoP	1.836	0.6	0.013	0.891
NF	0.233	24.0	0.021	0.963

Rs related to the series resistance. Rct denotes the charge transfer resistance. CPE is the constant phase angle element, which represents the double layer capacitance.

Table S5. Comparison over water splitting of performance for CoP/Fe-Co_9S_8 electrode with Co-based and Fe-based electrocatalysts in 1.0 M KOH.

Catalysts	j (mA/cm^2)	Potential (V)	Reference
CoP/Fe-Co_9S_8	10	1.448	**This work**
Fe-Co_9S_8	10	1.589	This work
CoP	10	1.632	This work
FeS_2/CoS_2	10	1.47	Small 2018, 14, 1801070.
Pt-CoS_2/CC	10	1.55	Adv Energy. Mater. 2018, 1800935.
$MoS_2/Co_9S_8/Ni_3S_2$/Ni	10	1.54	J. Am. Chem. Soc. 2019, 141, 10417-10430.
δ-FeOOH NSs/NF	10	1.62	Adv. Mater. 2018, 30, 1803144.
NiCoMo	10	1.43	ACS Energy Lett. 2019, 4, 952-959.
$Fe_{17.5\%}$-Ni_3S_2/NF	10	1.54	ACS Catal. 2018, 8, 5431.
$CoNiMoO_4$-21/CuO_x/CF	10	1.532	Adv. Energy Mater. 2021, 2102361
V-CoP	10	1.59	Adv. Energy Mater. 2021, 2101758
RuO_2/C || Pt/C	10	1.70	Nano Lett. 2020, 20, 136-144.

Table S6. The comparisons of the MEA performance of the CoP/Fe-Co_9S_8 at 1.6 V_{cell} with state-of-the-art examples in 1 M KOH.

**Anode**	**Cathode**	**Temperature /°C**	**Current density /mA·cm^{-2}**	**Reference**
$Li_xCo_{3-x}O_4$	Ni	45	300	*Int. J. Hydrog. Energy* 2013, 38, 3123-3129.
$Cu_{0.7}Co_{2.3}O_4$	Ni	55	300	*Int. J. Hydrog. Energy* 2012, 37, 9524-9528.
Ni foam	Ni foam	50	200	*J. Power Sources* 2016, 312, 128-136.
Ni foam	Ni foam	70	500	*J. Power Sources* 2017, 535, 45-55.
IrO_2	Pt/C	50	1000	*J. Power Sources* 2018, 382, 22-29
$NiCoO_x$:Fe	Pt/C	50	1000	*Adv. Energy Mater.* 2018, 8, 1801065.
$NiFe_2O_4$	NiFeCo alloy	50	500	*J. Power Sources* 2021, 481, 229093.
$NiMnO_x$	Pt/C	50	500	*Int. J. Hydrog. Energy* 2020, 45, 9285-9292
IrO_2	Pt/C	80	400	*J. Power Sources* 2020, 480, 228805.
$NiCo_2O_4$	$NiCo_2O_4$	50	400	*J. Mater. Chem. A* 2020, 8, 17089-17097.
VCoP	VCoP	60	1000	*Energy Environ. Sci.* 2022,15, 1882-1892
$Cu_{0.81}Co_{2.19}O_4$	Co_3S_4	50	1000	*Int. J. Hydrog. Energy,* 2020, 45, 36-45.
CoP/Fe-Co_9S_8	CoP/Fe-Co_9S_8	25	700	This work

References

[1] G. Kresse, J. Furthmüller, *Comput. Mater. Sci.* **1996**, *6*, 15.
[2] G. Kresse, J. Hafner, *Phys. Rev. B* **1994**, *49*, 14251.
[3] J. P. Perdew, K. Burke, M. Ernzerhof, *Phys. Rev. Lett.* **1996**, *77*, 3865.
[4] Y. Wang, A. Wang, Z. Xue, L. Wang, X. Li, G. Wang, *J. Mater. Chem. A* **2021**, *9*, 22597.
[5] X. Feng, Q. Jiao, Z. Dai, Y. Dang, S. L. Suib, J. Zhang, Y. Zhao, H. Li, C. Feng, A. Li, *J. Mater. Chem. A* **2021**, *9*, 12244.
[6] Y. Yang, H. Yao, Z. Yu, S. M. Islam, H. He, M. Yuan, Y. Yue, K. Xu, W. Hao, G. Sun, H. Li, S. Ma, P. Zapol, M. G. Kanatzidis, *J. Am. Chem. Soc.* **2019**, *141*, 10417.
[7] H. Xu, H. Jia, B. Fei, Y. Ha, H. Li, Y. Guo, M. Liu, R. Wu, *Appl. Catal. B: Environ.* **2020**, *268*.
[8] J. Li, Y. Tan, M. Zhang, W. Gou, S. Zhang, Y. Ma, J. Hu, Y. Qu, *ACS Energy Lett.* **2022**, *7*, 1330.
[9] T. Liu, P. Li, N. Yao, G. Cheng, S. Chen, W. Luo, Y. Yin, *Angew. Chem. Int. Ed. Engl.* **2019**, *58*, 4679.
[10] I. K. Mishra, H. Zhou, J. Sun, F. Qin, K. Dahal, J. Bao, S. Chen, Z. Ren, *Energy Environ. Sci.* **2018**, *11*, 2246.
